# Carrageenan Gum and Adherent Invasive *Escherichia coli* in a Piglet Model of Inflammatory Bowel Disease: Impact on Intestinal Mucosa-associated Microbiota

**DOI:** 10.3389/fmicb.2016.00462

**Published:** 2016-04-05

**Authors:** Peris M. Munyaka, Shadi Sepehri, Jean-Eric Ghia, Ehsan Khafipour

**Affiliations:** ^1^Department of Immunology, University of ManitobaWinnipeg, MB, Canada; ^2^Department of Animal Science, University of ManitobaWinnipeg, MB, Canada; ^3^Children Hospital Research Institute of ManitobaWinnipeg, MB, Canada; ^4^Section of Gastroenterology, Department of Internal MedicineWinnipeg, MB, Canada; ^5^Inflammatory Bowel Disease Clinical & Research Centre, University of ManitobaWinnipeg, MB, Canada; ^6^Department of Medical Microbiology, University of ManitobaWinnipeg, MB, Canada

**Keywords:** pigs, carrageenan gum, adherent invasive *Escherichia coli*, ulcerative colitis, Crohn's disease, 16S rRNA gene sequencing, microbiome

## Abstract

Inflammatory bowel diseases (IBD) including Crohn's disease (CD), and ulcerative colitis (UC), are chronic conditions characterized by chronic intestinal inflammation. Adherent invasive *Escherichia coli* (AIEC) pathotype has been increasingly implicated in the etiopathogenesis of IBD. In a 21-day study, we investigated the effects of AIEC strain UM146 inoculation on microbiota profile of the ileal, cecal, ascending and descending colon in a pig model of experimental colitis. Carrageenan gum (CG) was used to induce colitis in weaner piglets whereas AIEC strain UM146 previously isolated from a CD patient was included to investigate a cause or consequence effect in IBD. Treatments were: (1) control; (2) CG; (3) AIEC strain UM146; and (4) CG+UM146. Pigs in groups 2 and 4 received 1% CG in drinking water from day 1 of the study while pigs in groups 3 and 4 were inoculated with UM146 on day 8. Following euthanization on day 21, tissue mucosal scrapings were collected and used for DNA extraction. The V4 region of bacterial 16S rRNA gene was then subjected to Illumina sequencing. Microbial diversity, composition, and the predicted functional metagenome were determined in addition to short chain fatty acids profiles in the digesta and inflammatory cytokines in the intestinal tissue. CG-induced colitis decreased bacterial species richness and shifted community composition. At the phylum level, an increase in Proteobacteria and Deferribacteres and a decrease in Firmicutes, Actinobacteria, and Bacteroidetes were observed in CG and CGUM146 compared to control and UM146. The metabolic capacity of the microbiome was also altered in CG and CGUM146 compared to UM146 and control in the colon. We demonstrated that CG resulted in bacterial dysbiosis and shifted community composition similar to what has been previously observed in IBD patients. However, AIEC strain UM146 alone did not cause any clear changes compared to CG or control in our experimental IBD pig model.

## Introduction

Inflammatory bowel diseases (IBD), including Crohn's disease (CD), and ulcerative colitis (UC), are a group of inflammatory conditions related to the colon and small intestines and characterized by chronic inflammation. The etiopathology includes genetic susceptibility, environmental factors, deregulation of the immune system, and host relationship with commensal microbiota, as well as abnormal interactions between the intestinal microbiota and immune system (Abraham and Medzhitov, 2011; Khor et al., 2011; Maloy and Powrie, 2011; Manichanh et al., 2012). The intestinal microbiota plays a major role in gut development and in host homeostasis through development of immune regulation *via* inhibition of responses to inappropriate targets, such as gut contents and allergens, and by turning off inappropriate background inflammation (Hooper et al., 2002; Guarner, 2005). Therefore, abnormal shifts in the intestinal microbiota, termed dysbiosis, may lead to adverse health effects in the host, and could be critical in the pathogenesis and severity of IBD (Manichanh et al., 2006; Frank et al., 2007; Honda and Littman, 2012; Morgan et al., 2012; Fite et al., 2013). In this context, mice genetically modified for IBD do not develop colitis under germ-free conditions (Horwitz, 2007; Vijay-Kumar et al., 2007, 2010), and the therapeutic effect of antibiotics further supports bacterial contribution in the pathogenesis of IBD (Chapman et al., 1986; van Kruiningen, 1995; Vijay-Kumar et al., 2010).

A significant reduction in gut microbial diversity, which is characterized by a marked increase in the phylum Proteobacteria especially within Enterobacteriaceae family, or a decrease in the phylum Firmicutes (e.g., *Faecalibacterium prausnitzii*) has been observed in patients with IBD (Ott et al., 2004; Lupp et al., 2007; Sepehri et al., 2007; Sokol et al., 2008, 2009; Friswell et al., 2010; Qiu et al., 2013; Cao et al., 2014; Walters et al., 2014; Wright et al., 2015). Generally, the breakdown in the balance between “mutualistic and commensal” vs. “opportunistic and pathogenic” intestinal bacteria, largely characterized by reduced abundance of members of Firmicutes, Actinobacteria, and Bacteroidetes, and an increase in Proteobacteria and in some cases Bacteroidetes, is suggested to promote chronic intestinal inflammation (Sokol et al., 2008; Man et al., 2011; Walters et al., 2014; Wright et al., 2015).

Increased abundance of members of the Enterobacteriaceae family, especially *Escherichia coli*, have been observed in humans and dogs with IBD and also in experimental animal models of IBD (Sellon et al., 1998; Darfeuille-Michaud et al., 2004; Schuppler et al., 2004; Mylonaki et al., 2005; Kotlowski et al., 2007; Lupp et al., 2007; Xenoulis et al., 2008; Sepehri et al., 2009, 2011; Wright et al., 2015). Although, the adherent-invasive *E. coli* (AIEC) pathotype has been repeatedly identified in the intestinal mucosa of patients with CD (Darfeuille-Michaud et al., 1998, 2004; Agus et al., 2014), it is still difficult to pinpoint whether AIEC triggers intestinal inflammation leading to the disease, or if they colonize the gut mucosa as a consequence of pre-existing inflammation (Martin et al., 2004; Agus et al., 2014). Therefore, a cause and consequence relationship between *E. coli* strains and IBD is yet to be determined. To further investigate the role of AIEC in IBD, several AIEC strains, including LF82 and UM146, have been isolated from IBD patients and characterized (Miquel et al., 2010; Krause et al., 2011; Sepehri et al., 2011; Desilets et al., 2016). Strain UM146 was previously isolated in our lab from a CD patient and shown to be able to invade and replicate within macrophages, which is characteristic of an AIEC (Krause et al., 2011; Sepehri et al., 2011).

In the present study, we used a pig model of carrageenan gum (CG)-induced colitis to investigate the possible role of AIEC strain UM146 in IBD. Pigs share a similar gastrointestinal morphology and physiology with humans (Miller and Ullrey, 1987; Heinritz et al., 2013), which makes them more suitable model for human studies. CG, a sulfated polysaccharide, has been used in different animal models (mouse, guinea pig, rat, rabbit, rhesus monkey) to chemically induce intestinal ulceration or inflammation (Watt et al., 1979; Tobacman, 2001). The focus of these studies has been on the dynamic and profile of mucosal response in relation to CG-induced colitis and its similarity to that observed in IBD patients. However, there is no detailed and clear understanding of the structural and functional alterations of the intestinal microbiota in response to CG-induced colitis.

Here, we used Illumina high-throughput sequencing of the 16SrRNA gene and inferred metagenomics by PICRUSt to investigate differences in microbial composition and function in the ileum, cecum, ascending, and descending colon tissue samples of piglets with CG-induced colitis and inoculated with AIEC strain UM146. We demonstrated that CG-induced colitis influenced bacterial diversity and caused community composition changes at the phylum and lower taxonomical levels that are comparable to microbial changes observed in IBD patients. However, these changes were not observed in AIEC strain UM146 treatment. Similarly, the combination of CG and UM146 did not significantly influence the extent of the observed changes during CG-induced colitis.

## Materials and methods

### Animals and housing

A total of 24 male piglets [Duroc × (Yorkshire × Landrace)] weaned at 17 ± 2 d were obtained from Sunnyside Colony (Newton Siding, Manitoba, Canada). The pigs were housed in a temperature-controlled room within the T. K. Cheung Center for Animal Science Research, University of Manitoba (Winnipeg, MB, Canada). Room temperatures were maintained at 30°C during week (wk) 1 and 29°C during wk 2 and 3, with a 16 h lighting system. All pigs had *ad libitum* access to water and a basal diet in mash form formulated to meet or exceed the National Research Council (NRC, 2012) recommendations for a 7–11 kg pig. The experiment lasted for 21 days and the pigs were allowed to acclimate for 3 days before the start of experimental treatments.

### Ethical considerations

The procedures were approved by the Protocol Management and Review Committee of the University of Manitoba Animal Care Committee, and the pigs were cared for according to the guidelines of the Canadian Council of Animal Care (CCAC, 1993).

### Carrageenan gum

To induce mild ulcers on the intestinal tract of pigs, a 1% CG solution prepared from carrageenan powder (CarboMer, Inc., San Diego, CA, USA), a sulfated polysaccharide that induces predominantly mucosal and submucosal lesions with histological similarity to UC (Elson et al., 1995), was administered. The CG was administered via drinking water using elevated jugs connected to normal drinking nipples. The 1% concentration was chosen based on a pilot study conducted prior to this experiment (data not shown) where 0, 1, 2, and 4% CG concentrations were tested. Administration of 1% CG in drinking water only induced a mild injury in the pigs' gastrointestinal (GI) tract with varying degree of mucosa and sub mucosal edema, but without any granulomatous inflammation which is similar to what is observed in UC.

### Preparation of carrageenan gum

Undegraded CG has molecular weights of *M*_*n*_ 1.5 × 10^6^ to 2 × 10^7^ (Tong et al., 1980; Tobacman, 2001) and it is generally considered safe; however, degradation of CG to low molecular weights is associated with ulcerations and cancer-promoting effects (Watt et al., 1979; Tobacman, 2001). The solution used in this study was prepared by acid hydrolysis according to the procedure described previously (Watt et al., 1979), and is expected to yield a degraded carrageenan of average molecular weight of *M*_*n*_ 2 × 10^4^ to 3 × 10^4^ (Weiner, 1991; Marcus et al., 1992). Briefly, to each gram of the dry powder, one mL of concentrated HCl was added in a glass beaker and thoroughly mixed at room temperature (22°C) with a glass rod for 1 h. After 1 h, distilled water was added while stirring the mixture. The acidified solution was neutralized with 2 M sodium hydroxide to pH of 7–8 and the volume was adjusted to give a 1% concentration.

### Preparation of AIEC strain UM146

Adherent invasive *E. coli* (AIEC) strain UM146 previously isolated from a human subject with CD (Sepehri et al., 2011) was used in this study. The strain was cultured aerobically in LB broth and incubated at 37°C overnight to achieve a concentration of 10^8^–10^10^ CFU/mL, and 100 mL of the overnight culture was used for dosing as described below.

### Induction of colitis and inoculation with AIEC strain UM146

Pigs were weighed and randomly assigned to four treatment groups with three pigs per pen and two replicate pens per treatment. The treatments were as follows: (1) control; (2) pigs receiving 1% CG in drinking water; (3) pigs inoculated with AIEC strain UM146; and (4) as in 2 and 3; CGUM146. From day 1 of the study, pigs that were assigned to receive carrageenan gum were given a 1% CG solution in overhead plastic jugs connected to normal drinking water nipples. Administration of CG was done on a daily basis until end of the study (21 d). The CG solution level was monitored and jugs were refilled with freshly prepared CG every morning (at 9:30 am) and in the evening (at 4:30 pm), if needed. On day 8 of the study, pigs that were assigned to be inoculated with UM146 were given an overnight culture of the AIEC strain UM146 (Table 1). In this case, for each pig, 100 mL of the overnight culture was mixed with small amount of feed and the animals were allowed to consume the freshly mixed feed first.

**Table 1:** **Schedule of CG and adherent invasive *Escherichia coli* (AIEC) strain UM146 administration^a^**.

**Treatment administration**	**Study day**																					
	**Adaptation period (3 days)**	**1**	**2**	**3**	**4**	**5**	**6**	**7**	**8**	**9**	**10**	**11**	**12**	**13**	**14**	**15**	**16**	**17**	**18**	**19**	**20**	**21**
CG (1% in drinking water; *ad libitum*)		+	+	+	+	+	+	+	+	+	+	+	+	+	+	+	+	+	+	+	+	+
AIEC strain UM146									+													

a The first 3 days served as adaptation period. CG was administered from day 1 of the study until the end of the study, while administration of UM146 was done on day 8 only.

### Fecal score

Severity of diarrhea was characterized in a treatment-blinded manner by two trained individuals using a fecal consistency scoring system, as described previously (Marquardt et al., 1999). The scoring system was as follows: 0, normal; 1, soft feces; 2, mild diarrhea; and 3, severe diarrhea. Scoring was done at 48 h, 96 h, 7 d, and 14 d after inoculation with AIEC strain UM146.

### Tissue and digesta sampling

On days 21 and 22 of the study, all pigs were sedated by intramuscular injection of Ketamine:Xylazine (20:2 mg/kg BW) and euthanized by an intracardiac injection of 110 mg/kg BW sodium pentobarbital (Bimeda-MTC Animal Health Inc., Cambridge, ON, Canada). The abdominal cavity was opened from sternum to pubis to expose the gastrointestinal tract without damaging the wall of the digestive tract. The small intestine was stripped free of its mesentery and ileal, cecal, and colon digesta samples were obtained, divided into two sub-samples and transferred to sterile sample bags. One sub-sample of the digesta was used for determination of pH, whereas the second sub-sample was kept on ice and later transferred to −20°C for later analysis of volatile fatty acids (VFA). Tissue samples (two 5 cm long segments) were collected from the ileum, cecum, ascending and descending colon, flushed with sterile saline to remove excess lumen contents, immediately frozen in liquid nitrogen and transferred to −80°C until used for DNA extraction and further molecular/microbial analyses.

### Analysis of pH, ammonia N, and volatile fatty acids

pH was measured immediately after digesta collection using an Accumet Basic 15 pH meter (Fisher Scientific, Fairlawn, NJ) equipped with a Sensorex 450C Flat Surface Combination pH/Reference Electrode (Sensorex, Stanton, CA), which was standardized with certified pH 4 and 7 buffer solutions. Ammonia nitrogen concentration was measured using a calorimetric technique as described previously (Novozamsky et al., 1974), while volatile fatty acids (VFA) were determined using gas chromatography (Bhandari et al., 2007).

### Characterization of inflammatory responses

Tissue samples were homogenized (50 mg/mL) in Tris lysis buffer (Meso scale discovery diagnostics, Rockvile, MD, USA) containing protease inhibitors (Roche, Mississauga, ON, Canada). Samples were centrifuged at 3000 × *g* for 10 min and the supernatant was recovered and stored at −80°C until analyzed. Cytokine levels [interleukin (IL)-1β, IL-6, IL-8, IL-10, and tumor necrotic factor (TNF)-α] were determined using a custom meso scale porcine kit (Meso scale discovery diagnostics, Rockville, MD, USA), according to the manufacturer instructions.

### Correlation coefficients

Associations between bacterial taxa with an abundance ≥0.5% of community in the ileum, cecum, and ascending colon and short chain fatty acids (acetate, propionate, butyrate), or inflammatory markers (IL-1β, IL-6, IL-8, IL-10, TNF-α) were explored using non-parametric Spearman's rank correlation implemented in PAST software (Hammer et al., 2001). For each correlation, correlation coefficient (Spearman's Rho) and *P*-value were obtained and the resulting correlation matrix was visualized in a heatmap format generated by the corrplot package of R (Corrplot: visualization of a correlation matrix. R package ver. 02-0.2010; http://CRAN). The correlation coefficient values ranged from −1 to +1 with larger absolute values indicating stronger relationship while positive and negative values indicating the direction of association. Alpha value for the correlation confidence intervals was set up as 0.05.

### DNA extraction

Tissue samples were thawed at room temperature. The inner wall was then gently scrapped with a blunt blade to obtain 200–300 mg of mucosa, of which, ~50 mg was used for DNA extraction. DNA was extracted using a ZR Tissue and Insect DNA kit (Zymo Research Corp., Orange, CA), which included a bead-beating step for the mechanical lysis of the microbial cells. DNA concentration was determined using a NanoDrop 2000 spectrophotometer (ThermoFisher Scientific, Wilmington, DE, USA), and the DNA quality was evaluated by PCR amplification of the 16S rRNA gene using universal primers 27F (5′-GAAGAGTTTGAT CATGGCTCAG-3′) and 342R (5′-CTGCTG CCTCCCGTAG-3′), as previously described (Khafipour et al., 2009). Amplicons were verified using agarose gel electrophoresis.

### Library construction and illumina sequencing

The V4 region of 16S rRNA gene was targeted for PCR amplification using modified F515/R806 primers (Caporaso et al., 2012), as previously described (Derakhshani et al., 2016). Briefly, the reverse PCR primer was indexed with 12-base Golay barcodes allowing for multiplexing of samples. The PCR reaction for each sample was performed in duplicate and contained 1.0 μL of pre-normalized DNA (20ng/μL), 1.0 μL of each forward and reverse primers (10 μM), 12 μL HPLC grade water (Fisher Scientific, Ottawa, ON, Canada), and 10 μL 5 Prime Hot MasterMix (5 Prime, Inc., Gaithersburg, MD, USA). Reactions consisted of an initial denaturing step at 94°C for 3 min followed by 35 amplification cycles at 94°C for 45 s, 50°C for 60 s, and 72°C for 90 s, and an extension step at 72°C for 10 min in an Eppendorf Mastercycler pro (Eppendorf, Hamburg, Germany). PCR products were then purified using ZR-96 DNA Clean-up Kit™ (ZYMO Research, Irvine, CA, USA) to remove primers, dNTPs, and reaction components. The V4 library was then generated by pooling 200 ng of each sample and quantified using Picogreen (Invitrogen, Burlington, NY, USA). This was followed by multiple dilution steps using pre-chilled hybridization buffer (HT1; Illumina, San Diego, CA, USA) to bring the pooled amplicons to a final concentration of 5 pM, measured by Qubit 2.0 Fluorometer (Life technologies, Burlington, ON, Canada). Finally, 15% of PhiX control library was spiked into the amplicon pool to improve the unbalanced and biased base composition, a known characteristic of low diversity 16S rRNA libraries. Customized sequencing primers for read1 (5′-TATGGTAATTGTGTG CCAGCMGCCGCGGTAA-3′), read2 (5′-AGT CAGTCAGCCGGACTACHVGGGTWTCTA AT-3′), and index read (5′-ATTAGAWACCCBDGT AGTCCGGCTGACTGACT-3′; Integrated DNA Technologies, Coralville, IA, USA) were added to the MiSeq Reagent V2 Kit (300-cycle; Illumina, San Diego, CA, USA). The 150 paired-end sequencing reaction was performed on a MiSeq platform (Illumina, San Diego, CA, USA) at the Gut Microbiome and Large Animal Biosecurity Laboratories (Department of Animal Science, University of Manitoba, Winnipeg, MB, Canada). The sequencing data were uploaded into the Sequence Read Archive (SRA) of NCBI (http://www.ncbi.nlm.nih.gov/sra) and can be accessed through accession number SRR2601043.

### Bioinformatic analyses

The PANDAseq assembler (Masella et al., 2012) was used to merge overlapping paired-end Illumina fastq files. All the sequences with low quality base calling scores as well as those containing uncalled bases in the overlapping region were discarded. The output fastq file was then analyzed by downstream computational pipeline of the open source software package QIIME (Caporaso et al., 2010b). Assembled reads were demultiplexed according to the barcode sequences and chimeric reads were filtered using UCHIME (Edgar et al., 2011) and sequences were assigned to Operational Taxonomic Units (OTU) using the QIIME implementation of UCLUST (Edgar, 2010) at 97% pairwise identity threshold using an open-reference OTU picking process (Rideout et al., 2014). Taxonomies were assigned to the representative sequence of each OTU using RDP classifier (Wang et al., 2007) and aligned with the Greengenes (v. 13.5) reference database (DeSantis et al., 2006) using PyNAST algorithms (Caporaso et al., 2010a). Phylogenetic tree was built with FastTree 2.1.3. (Price et al., 2010).

### Alpha- and beta-diversities

Within-community diversity (α-diversity) was calculated by different indices of species richness and evenness including observed number of species, Chao1, abundance-based coverage estimator (ACE), Shannon, Simpson, Inverse Simpson (InvSimpson), and Fisher using the open source R software (3.1.0). The *P*-values were calculated using the SAS MIXED procedure (SAS 9.3). An even depth of 5000, 4000, 12,000, and 10,000 sequences per sample was used to calculate the richness and diversity indices for the ileum, cecum, ascending, and descending colon, respectively. To assess the beta-diversity (β-diversity) differences among bacterial communities from different treatments within each compartment, non-metric multidimensional scaling (nMDS) ordination plots were generated using R software (3.1.0) by employing Bray-Curtis similarity matrices with a conventional cut-off of < 0.2 for the stress value. The resulting minimum stress solution was used to produce the nMDS plots, in which each data point represents one sample. The spatial distance between points in the plot was interpreted as the relative difference in the bacterial community composition; thus, points that were closer were more similar than points that were more distant. To assess the statistical differences in β-diversity of bacterial communities among treatment groups, permutation multivariate analysis of variance (PERMANOVA; Anderson, 2005) was performed and *P*-values were calculated.

### Partial least square discriminant analysis

Partial least square discriminant analysis (PLS-DA; SIMCA P+ 13.02, Umetrics, Umea, Sweden) was performed on the lower taxonomic data to identify the effects of CG and UM146 (Li et al., 2012). The PLS-DA is a particular case of partial least square regression analysis in which Y is a set of variables describing the categories of a categorical variable on X. In this case, X variables were bacterial genera and Y was observations of different treatments compared together. To avoid over-parameterization of the model, variable influence on projection value (VIP) was estimated for each taxa, and taxa with VIP < 0.5 were removed from the final model. *R*^2^ estimates were used to evaluate the goodness of fit and *Q*^2^ estimate was used to evaluate the predictive value of the model. Data are presented in loading scatter plots. The PLS-DA regression coefficients were used to identify taxa that were positively or negatively correlated with each treatment group. The significant shifts of taxa were determined when the error bars of each component was above or below x axis of coefficient plot (Wang et al., 2016).

### Prediction of functional metagenomics

The open source software PICRUSt (Phylogenetic Investigation of Communities by Reconstruction of Unobserved States; v. 1.0.0-dev) was used to predict the functional capacity of microbiome using 16S rRNA gene sequencing data and Greengenes (v. 13.5) reference database (DeSantis et al., 2006). To make our open-reference picked OTUs compatible with PICRUSt, all *de-novo* OTUs were removed and only those that had matching Greengenes identifications were retained. The new OTU table was then used to generate metagenomic data after normalizing the data by copy numbers, and to derive relative Kyoto Encyclopedia of Genes and Genomes (KEGG) pathway abundance (Langille et al., 2013). The KEGG data was analyzed using STAMP (STatistical Analysis of Metagenomic Profiles (Parks and Beiko, 2010). To determine the functional KEGG pathways that could be associated with the microbial changes observed, we compared the functional pathways for the mucosal microbiota of samples from CG and CGUM146 to control or UM146 at all the intestinal sites (ileum, cecum, ascending and descending colon).

### Other statistical analysis

For the fecal score, cytokines, VFAs, pH, ammonia, and the phylum data, the treatment effect was evaluated using a completely randomized design and the data was subjected to ANOVA using the MIXED procedure of SAS (v. 9.3). Differences between means were determined using Tukey's test. SAS UNIVARIATE procedure was used to test the normality of residuals. For non-normally distributed data, Poisson and negative binomial distributions were fitted in GLIMMIX procedure of SAS and the goodness of fit for different distributions was determined using Pearson chi-square /DF ratio (closer to 1 is better). In both MIXED and GLIMMIX models, the effect of treatment was considered fixed and pig were treated as random factor. The differences between treatments were considered significant at *P* < 0.05.

## Results

### Diarrhea post-inoculation with AIEC strain UM146

**Figure 1:**
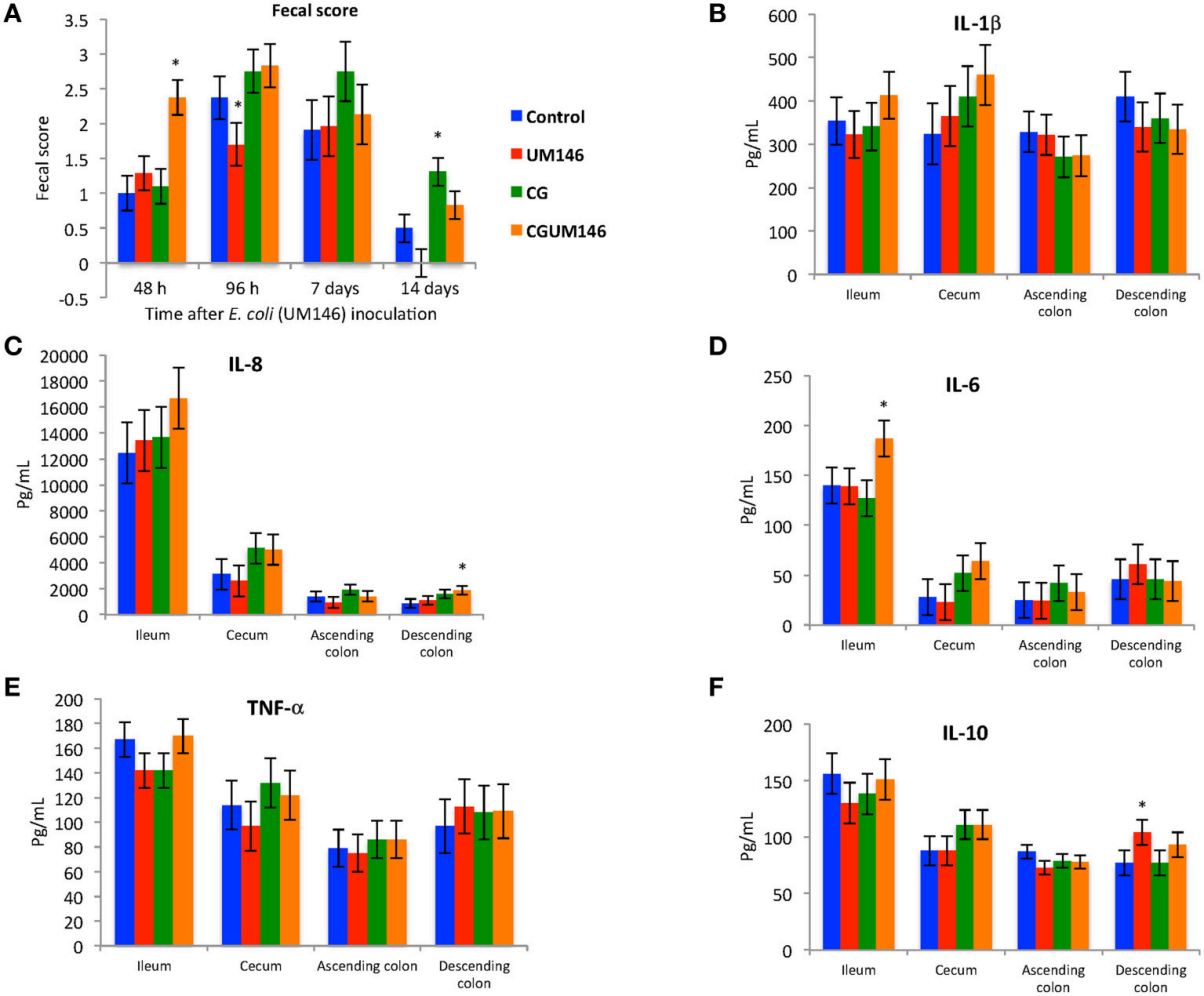
**(A–F)** The effect of carrageenan gum (CG) and adherent invasive *Escherichia coli* (AIEC) strain UM146 inoculation on fecal score, as determined by stool consistency, and on inflammatory markers (IL-1β, IL-6, IL-8, IL-10, and TNF-α) in weaned pigs. CG was administered to the designated groups from day 1 of the experiment. CG: pigs received 1% carrageenan gum only in drinking water daily; UM146: pigs were inoculated with AIEC UM146 on day 8; CGUM146: pigs received CG from day 1 and were inoculated with AIEC UM146 on day 8. Pigs in designated groups received 100 mL of AIEC UM146 culture (10^8^ CFU/mL) in feed. Severity of diarrhea was characterized using an established fecal consistency (FC) score system in pigs (0, normal; 1, soft feces; 2, mild diarrhea; 3, severe diarrhea). ^*^Designates a significant difference compared to other groups; *P* < 0.05.

At 48 h post-inoculation, diarrhea was observed in colitic pigs that were also inoculated with *E. coli* (CGUM146 treatment) but not in the other treatment groups (Figure 1A). At 96 h post-inoculation, pigs in UM146 treatment had soft feces (an average fecal score of 1.5) compared to pigs in other treatments that experienced mild diarrhea (an average fecal score of 2.5).

### Volatile fatty acids, ammonium N, and pH in the ileum, cecum and colon

As shown in Table 2, ammonium N and pH were similar among treatments in the ileum, cecum and colon except for a lower ammonium N in the cecum that was observed in the UM146 treatment compared to the control. VFA concentrations were not different among treatment groups in the ileum. In both cecum and colon, butyrate was significantly lower in the CG treatment compared to UM146, but the difference wasn't significant compared to other treatments. In addition, colonic acetate was significantly lower in both CG and CGUM146 treatments compared to the control, but there was no significant difference compared to the UM146 treatment.

**Table 2:** **Effect of carrageenan gum (CG)^1^ and adherent invasive *Escherichia coli* (AIEC) strain UM146^2^ inoculation on microbial activities in the ileum, cecum and colon digesta of weaned pigs, as determined by changes in the level of pH, ammonium nitrogen (N), and volatile fatty acids (VFA)**.

**Item**	**Treatment^3^**				**SED^4^**	***P*-value^5^**
	**Control**	**CG**	**UM146**	**CGUM146**		
**pH**						
Ileum	6.00	6.06	5.62	6.20	0.37	0.4667
Cecum	5.63	5.59	5.55	5.68	0.17	0.9044
Colon	5.84	6.01	5.82	5.84	0.15	0.6118
**AMMONIUM N, mg/dL**						
Ileum	23.86	23.62	19.13	19.97	3.4	0.4165
Cecum	17.01^a^	14.43^a^^b^	10.51^b^	14.61^a^^b^	2.3	0.0729
Colon	35.95	30.83	30.75	27.83	4.7	0.4113
**ILEUM VFA, mmol/mL**						
Acetate	8.17	6.47	4.33	6.57	1.48	0.1590
Propionate	0.25	0.23	0.11	0.30	0.11	0.4697
Butyrate	0.39	0.04	0.69	0.37	0.35	0.3823
Valerate	0.29	0.21	0.22	0.27	0.09	0.7313
Iso-butyrate	0.49	0.37	0.28	0.28	0.4	0.9473
Iso-valerate	0.48	0.33	0.42	0.38	0.16	0.8369
**CECAL VFA, mmol/mL**						
Acetate	48.6	38	56	45.54	6.8	0.1017
Propionate	24.86	28	32.37	26.12	4.4	0.3785
Butyrate	13.94^a^^b^	8.9^b^	21.42^a^	18.58^a^^b^	4.1	**0.0352**
Valerate	3.52	2.39	6.09	6.55	1.7	0.0695
Iso-butyrate	0.12	0.20	0.32	0.23	0.09	0.2715
Iso-valerate	0.31	0.35	0.55	0.51	0.15	0.3391
**COLON VFA, mmol/mL**						
Acetate	54.88^a^	42.12^b^	50.34^a^^b^	42.35^b^	4.3	**0.0185**
Propionate	24.39	24.69	27.04	20	3.3	0.2390
Butyrate	14.91^a^^b^	9.75^b^	17.06^a^	13.79^a^^b^	1.9	**0.0114**
Valerate	4.37	3.36	5.17	4.86	1.02	0.3377
Iso-butyrate	0.3	0.14	0.34	0.21	0.08	0.1023
Iso-valerate	0.81	0.67	0.80	0.64	0.18	0.7186

a,b Means with different superscripts within the same row differs significantly; P < 0.05.

1 Carrageenan gum (CG) was administered in the designated groups from day 1 of the experiment. Pigs received 1% CG in drinking water on daily basis.

2 AIEC UM146 inoculation was administered on day 8. Pigs in designated groups received 100 mL of an overnight AIEC UM146 culture (10^8^ CFU/mL) in feed.

3 CG: pigs received 1% CG only in drinking water daily; UM146: pigs were inoculated with AIEC UM146 on day 8; CGUM146: pigs received CG from day 1 and were inoculated with AIEC UM146 on day 8.

4 SED: standard error of difference between treatment means.

5 Significant P-values are highlighted with bold font.

### Inflammatory cytokines in the ileum, cecum, ascending, and descending colon

Among the cytokines analyzed in the four intestinal segments on day 21, CGUM146 treatment up-regulated IL-8 and IL-6 in the descending colon and ileum, respectively, whereas UM146 up-regulated IL-10 in the descending colon. IL-1β and TNF-α did not differ among treatment groups in all intestinal segments examined (Figures 1B–F).

### Correlation coefficient

As shown in Table 3 and Supplementary Figures 1–3, several taxa were positively or negatively correlated with various short chain fatty acids and/or inflammatory markers in the ileum, cecum and ascending colon.

**Table 3:** **Alpha-diversity indices of the ileal, cecal, ascending, and descending colon mucosa-associated microbiota of pigs treated with carrageenan gum (CG)^1^ and inoculated with adherent invasive *Escherichia coli* (AIEC) strain UM146^2^**.

**Items**	**Treatment^3^**				**SED^4^**	***P*-value^5^**
	**Control**	**UM146**	**CG**	**CGUM146**		
**ILEUM**						
Observed species	95.16	100.25	105.70	147.17	36.20	0.43
Chao1	176.75	163.61	179.67	245.36	57.40	0.54
ACE	191.20	170.51	202.32	263.20	68.30	0.56
Shannon	1.71	1.76	1.98	2.64	0.60	0.45
Simpson	0.58	0.48	0.60	0.76	0.15	0.40
InvSimpson	2.93	2.63	2.59	5.35	1.10	0.06
Fisher	22.14	25.93	24.13	37.80	10.20	0.47
**CECUM**						
Observed species	442.29^a^	422.33^a^	255.71^b^	337.50^a^^b^	68.50	**0.05**
Chao1	765.11^a^	736.57^a^	439.83^b^	623.27^a^^b^	124.00	**0.07**
ACE	845.64^a^	790.69^a^	482.55^b^	576.43^a^^b^	115.00	**0.01**
Shannon	3.50	3.47	2.42	2.91	0.47	0.11
Simpson	0.90^a^	0.92^a^	0.70^b^	0.80^a^^b^	0.07	**0.02**
InvSimpson	13.18^a^	13.54^a^	4.00^b^	5.40^b^	3.40	**0.01**
Fisher	120.14^a^	122.11^a^	58.25^b^	84.91^a^^b^	20.80	**0.02**
**ASCENDING COLON**						
Observed species	912.43^a^	807.33^a^^b^	574.00^b^	537.22^b^	126.00	**0.01**
Chao1	1597.16^a^	1329.93^a^	985.36^a^^b^	783.04^b^	268.00	**0.02**
ACE	1737.07^a^	1425.97^a^^b^	1049.47^a^^b^	826.98^b^	296.00	**0.02**
Shannon	4.25^a^	4.20^a^^b^	3.12^b^	3.59^a^^b^	0.04	0.05
Simpson	0.93^a^	0.925^a^	0.77^b^	0.88^a^^b^	0.06	**0.04**
InvSimpson	21.45^a^	18.210^a^	5.15^b^	10.14^a^^b^	4.70	**<0.01**
Fisher	242.25^a^	201.67^a^^b^	137.39^b^	121.39^b^	27.80	**0.02**
**DESCENDING COLON**						
Observed species	1012.29^a^	989.67^a^	652.14^b^	680.29^b^	143.00	**0.02**
Chao1	1800.90	1726.70	1195.66	1180.20	332.00	0.13
ACE	1942.05	1928.19	1280.03	1331.88	360.00	0.14
Shannon	0.97^a^	0.95^a^	0.86^b^	0.90^a^^b^	0.03	**<0.01**
Simpson	289.74	280.52	165.38	176.30	47.90	**0.02**
InvSimpson	40.75^a^	28.54^a^^b^	13.39^b^	12.61^b^	6.00	**<0.01**
Fisher	289.74^a^	280.52^a^^b^	165.38^b^	176.30^a^^b^	47.80	**0.02**

a,b Means with different superscripts within the same row differs significantly; P < 0.05.

1 Carrageenan gum was administered to the designated groups from d 1 of the experiment. Pigs received 1 % of CG in drinking water on daily basis.

2 AIEC UM146 inoculation was administered on d 8 of the experiment. Pigs in designated groups received 100 mL of an overnight AIEC UM146 culture (10^8^ CFU/mL) in feed.

3 CG = pigs received 1% carrageenan gum only in drinking water on daily basis, UM146 = pigs were inoculated with AIEC UM146 on d 8 of the study, CGUM146 = pigs received CG from d 1 of the study and were inoculated with AIEC UM146 on d 8 of the study.

4 SED: standard error of difference between treatment means.

5 Significant P-values are highlighted with bold font.

### Alpha-diversity in different intestinal segments

As shown in Table 3, no significant difference was observed among treatment groups in the ileum. Also, according to most of the diversity indices calculated, lower bacterial diversity was observed in both CG and CGUM146 treatments compared to control and UM146 in the cecum, ascending and descending colon.

### Beta-diversity differences among treatment groups in the ileum, cecum, ascending, and descending colon

**Figure 2:**
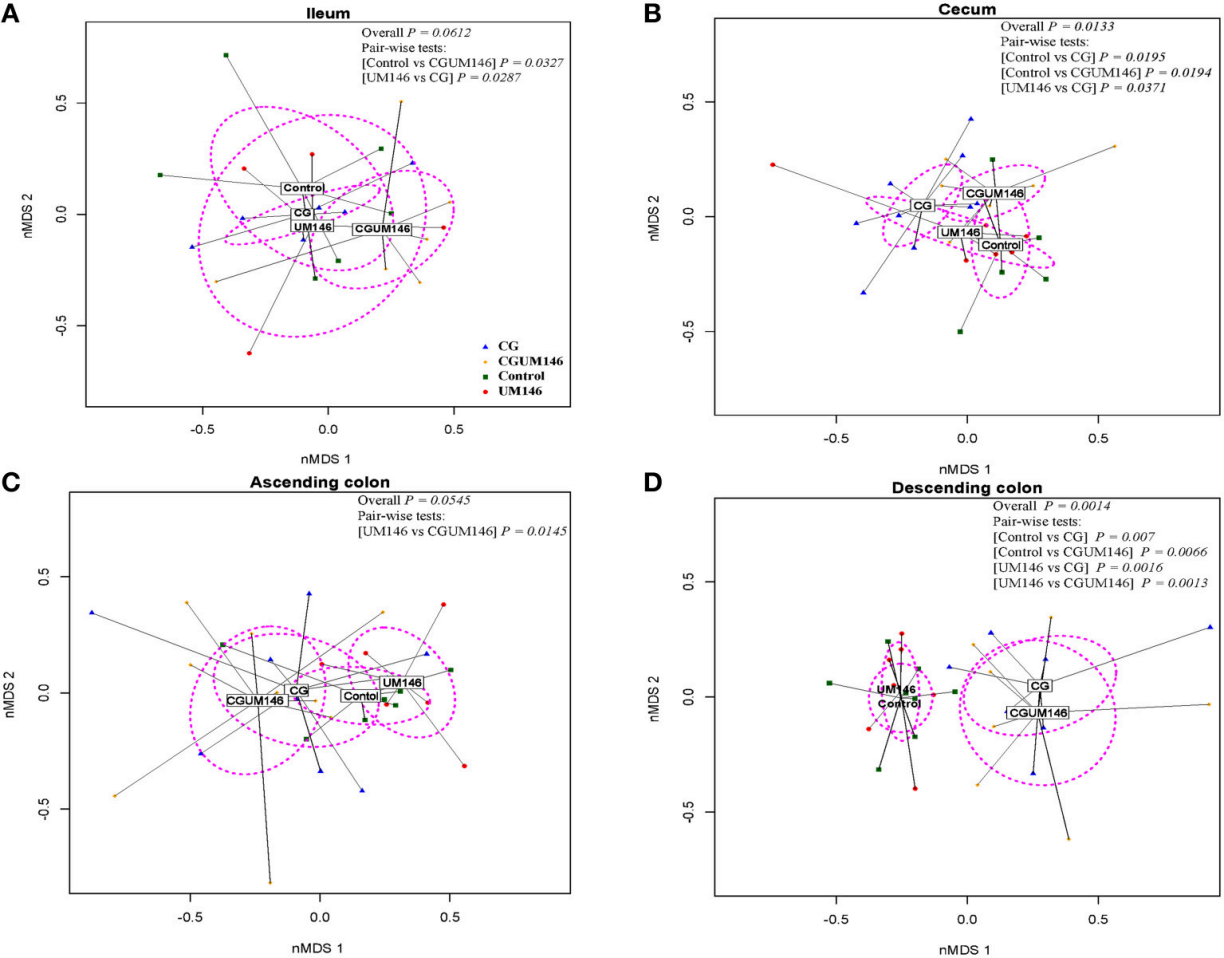
**Non-metric multidimensional scaling (nMDS) ordination plot, a measure of relative difference in the bacterial community composition in the: (A) ileum; (B) cecum; (C) ascending colon; and (D) descending colon of pigs treated with carrageenan gum (CG) and inoculated with adherent invasive *Escherichia coli* (AIEC) strain UM146**. The colored points are shaded according to different treatment groups. CG: pigs received 1% carrageenan gum only in drinking water daily; UM146: pigs were inoculated with AIEC UM146 on day 8 of the study; CGUM146: pigs received CG from day 1 of the study and were inoculated with AIEC UM146 on day 8 of the study. Pigs in designated groups received 100 mL of AIEC UM146 culture (10^8^ CFU/mL) in feed. The *P*-values were calculated using PERMANOVA. For the pair-wise tests, only the significant *P*-values are included.

As shown in Figures 2A–D, PERMANOVA analysis of beta-diversity data showed significant difference between treatment groups in the cecum (*P* = 0.013) and descending colon (*P* = 0.0014). In this context, cecal, and descending colon samples clustered separately according to treatment status of the pig, suggesting that the samples are composed of distinct bacterial communities.

In the ileum and ascending colon although overall *P*-values were not significant (ileum, *P* = 0.0612; ascending colon, *P* = 0.0545) pair-wise comparisons between control vs. CGUM146 (*P* = 0.0327) and UM146 vs. CG (*P* = 0.0287) in the ileum, and UM146 vs. CGUM146 (*P* = 0.0145) in the ascending colon were significant.

### Microbiota composition at the phylum and lower taxonomic levels in different intestinal segments

#### Ileum

**Figure 3:**
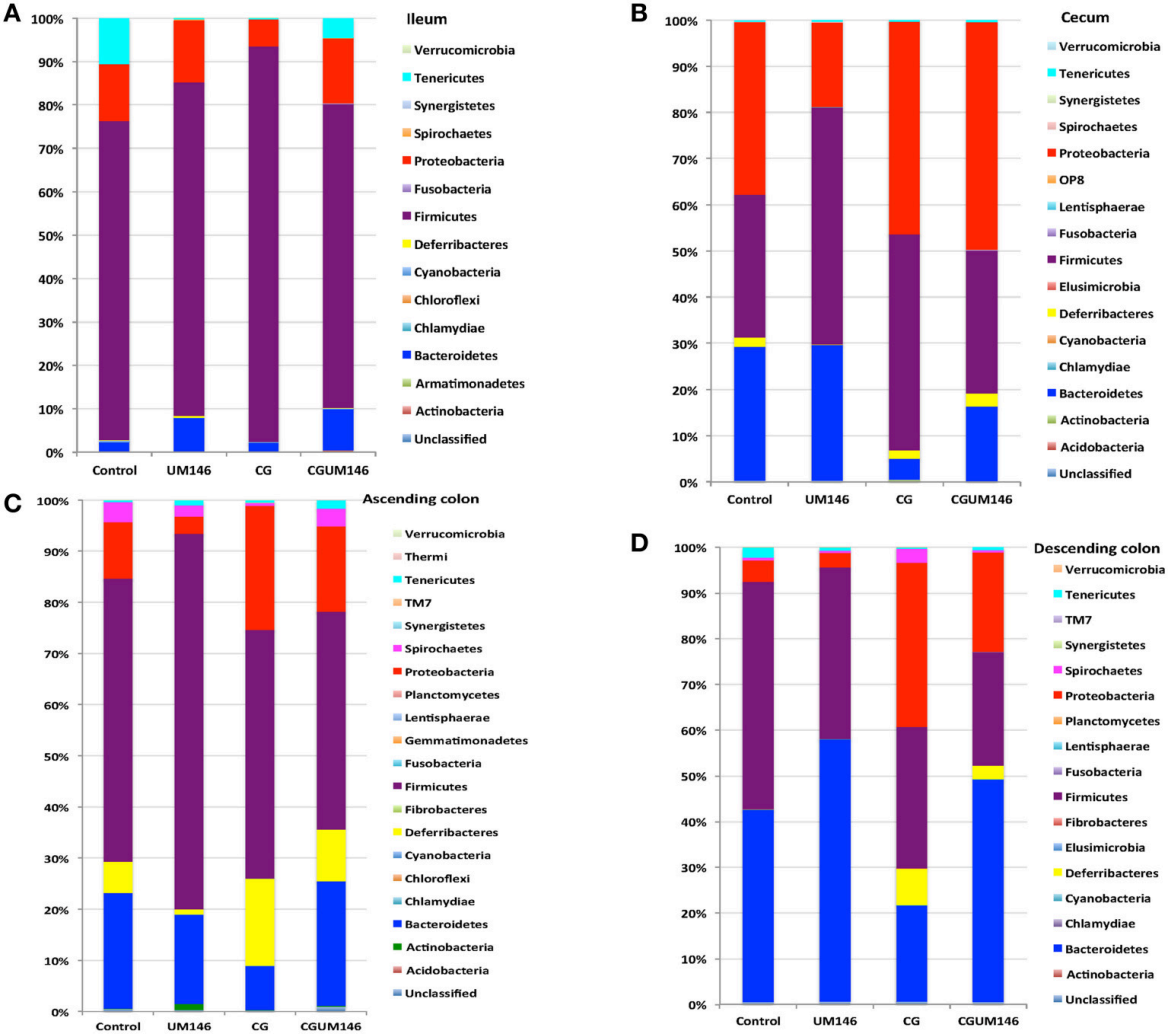
**Percentage of relative abundance of bacterial phyla in the: (A) ileum; (B) cecum; (C) ascending colon; and (D) descending colon of pigs treated with carrageenan gum (CG) and inoculated with adherent invasive *Escherichia coli* (AIEC) strain UM146. (A) Ileum**: A total of 14 phyla were identified in all the samples, of which four were considered to be abundant within the community (≥1%), including Firmicutes, Bacteroidetes, Proteobacteria, and Tenericutes. Among the abundant phyla, Firmicutes was the most dominant phylum but its proportion did not differ among treatment groups. **(B) Cecum**: A total of 16 phyla were identified in all the samples, of which four were considered to be abundant within the community (≥1%), including Firmicutes, Bacteroidetes, Proteobacteria, and Deferribacteres. Among these abundant phyla, the proportion of Proteobacteria was slightly higher (*P* < 0.05) in the CG and CGUM146 groups compared to the control and UM146 treatments, whereas Bacteroidetes was lower (*P* < 0.05) in the CG and CGUM146 groups compared to the control and UM146 treatments. **(C) Ascending colon**: A total of 20 phyla were identified in all the samples, of which five were considered to be abundant within the community (≥1%), including Firmicutes, Bacteroidetes, Proteobacteria, Deferribacteres, and Spirochaetes. The proportion of Firmicutes was lower in CG and CGUM146 groups (*P* = 0.007), while Proteobacteria (*P* = 0.001) and Deferribacteres (*P* = 0.039) were higher in these groups compared to control and UM146 treatments. Bacteroidetes abundance was lower (*P* < 0.0001) in CG treatment compared to other groups whereas Spirochaetes did not differ among groups (*P* > 0.05). **(D) Descending colon**: A total of 17 phyla were identified in all the samples, of which five were considered to be abundant within the community (≥1%), including Firmicutes, Bacteroidetes, Proteobacteria, Deferribacteres, and Tenericutes. The proportion of Firmicutes was lower (*P* = 0.0055) in the CG and CGUM146 treatments while Proteobacteria proportion was higher (*P* < 0.0001) in these groups compared to the control and UM146 treatments. The Bacteroidetes proportion was lower (*P* < 0.0001) in CG treatment compared to other treatment groups. CG: pigs received 1% CG only in drinking water daily; UM146: pigs were inoculated with AIEC UM146 on day 8; CGUM146: pigs received CG from day 1 and were inoculated with AIEC UM146 on day 8. Pigs in designated groups received 100 mL of AIEC UM146 culture (10^8^ CFU/mL) in feed.

**Figure 4:**
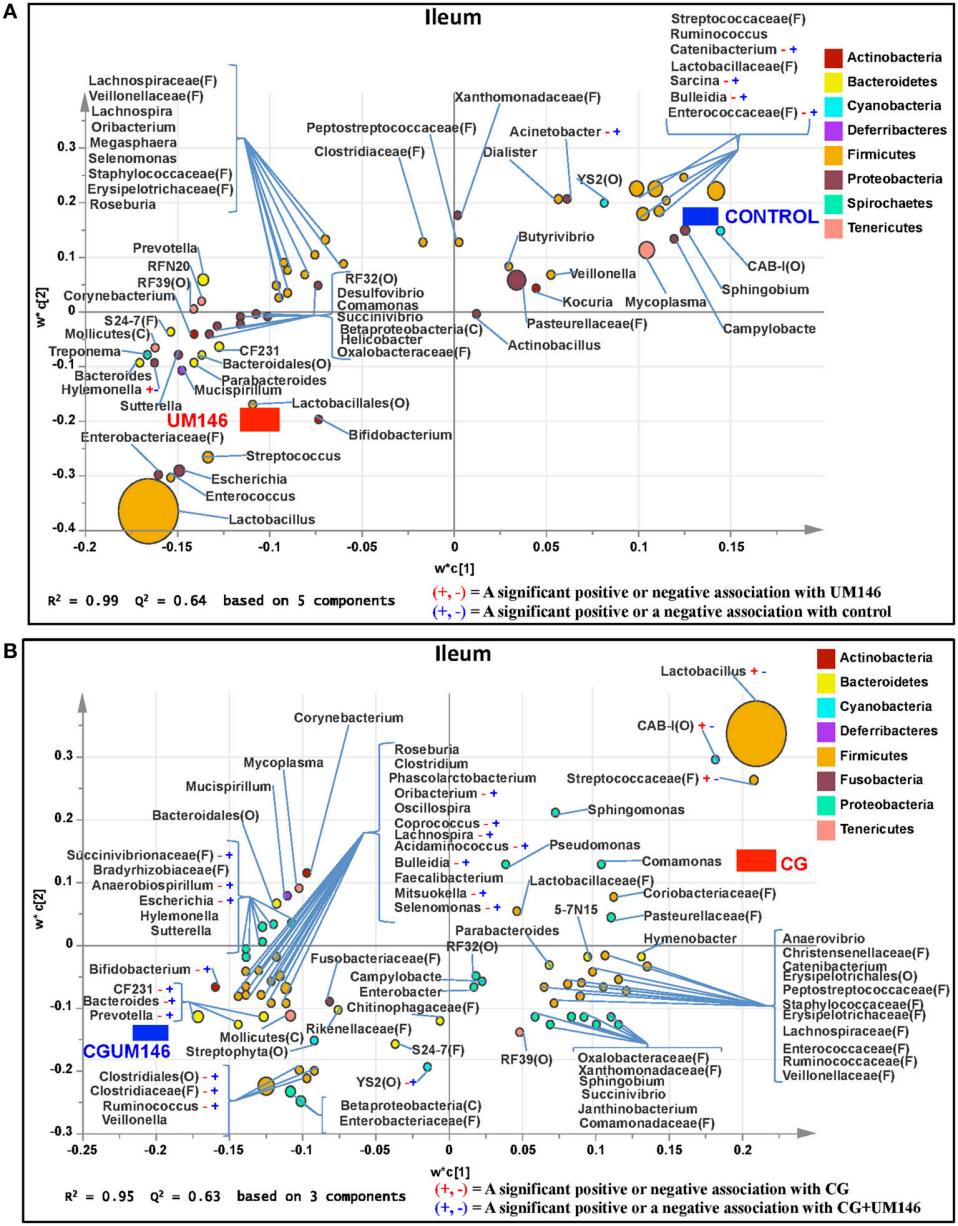
**Taxa that were positively or negatively associated with: (A) control or UM146 in the ileum of pigs inoculated with adherent invasive *Escherichia coli* (AIEC) strain UM146; (B) CG or CGUM146 in the ileum of pigs treated with CG and inoculated with AIEC UM146**. UM146: pigs were inoculated with AIEC UM146 on day 8; CG: pigs received 1% CG only in drinking water daily; CGUM146: pigs received CG from day 1 and were inoculated with AIEC UM146 on day 8. Pigs in designated groups received 100 mL of AIEC UM146 culture (10^8^ CFU/mL) in feed.

A total of 266,412 quality-filtered sequences were obtained from the samples with an average of 11,583 sequences per sample. Fourteen phyla were identified in all the samples. Among the most abundant phyla, Firmicutes was the most dominant phylum but the phyla did not differ significantly between treatment groups (Figure 3A, Supplementary Table 1.) Classification of OTUs at lower taxonomical levels resulted in the identification of 191 taxa of which 95 were ≥0.01% of the community, while 96 taxa were < 0.01% of the community. Bacterial taxa with relative abundance of >0.01% of community were analyzed using PLS-DA to identify taxa that were most characteristic of different treatment groups and the results are shown in Figures 4A,B. Supplementary Table 2 shows a summary of the mean abundance of all the taxa.

#### Cecum

**Figure 5:**
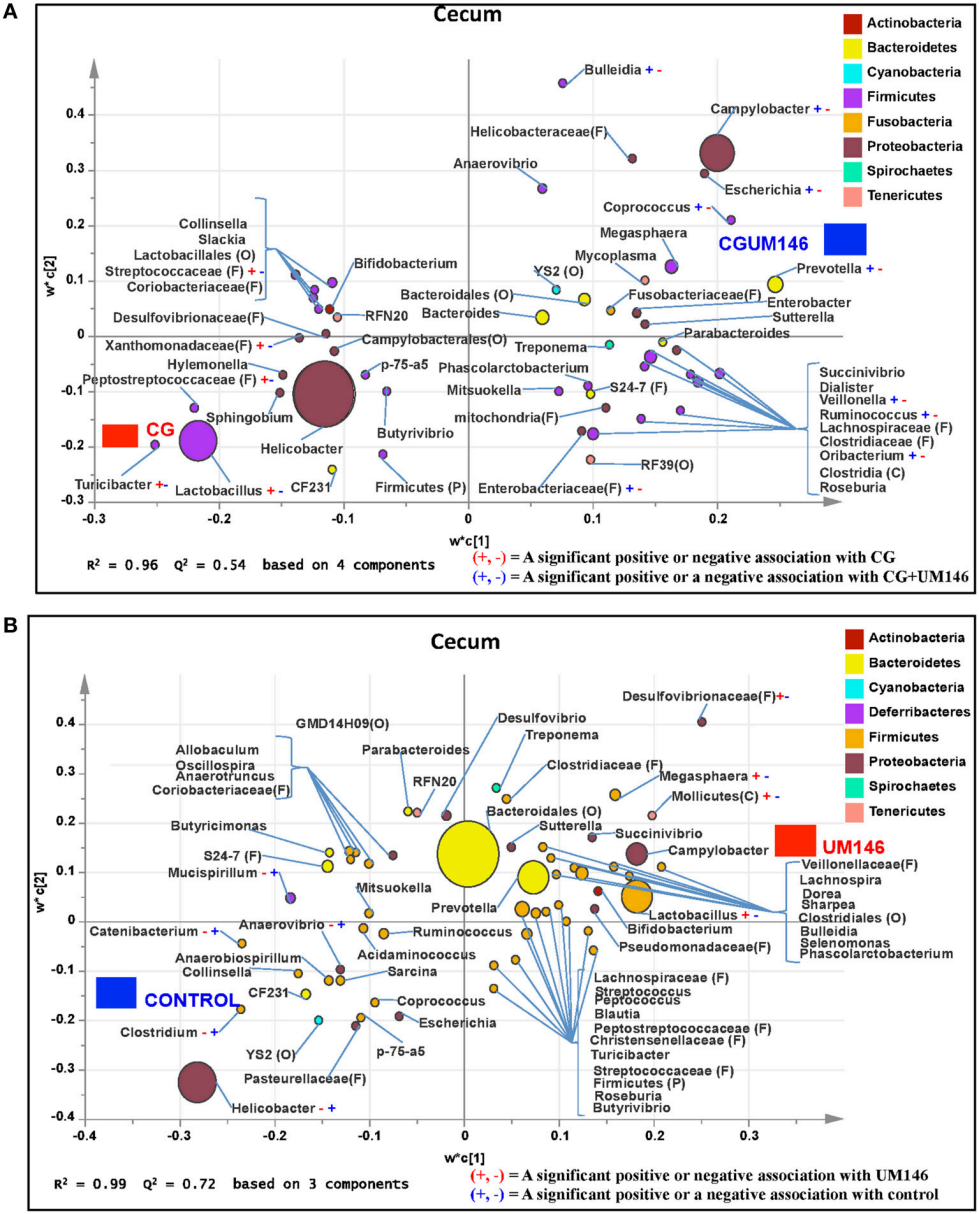
**Taxa that were positively or negatively associated with: (A) CG or CGUM146 in the cecum of pigs treated with CG and inoculated with adherent invasive *Escherichia coli* (AIEC) strain UM146; (B) control or UM146 in the cecum of pigs inoculated with AIEC UM146**. CG: pigs received 1% CG only in the drinking water daily; CGUM146: pigs received CG from day 1 and were inoculated with AIEC UM146 on day 8; UM146: pigs were inoculated with AIEC UM146 on day 8. Pigs in designated groups received 100 mL of AIEC UM146 culture (10^8^ CFU/mL) in feed.

A total of 571,706 quality-filtered sequences were obtained from the samples with a mean of 19,056 sequences per sample. Sixteen phyla were identified in all the samples. Among the most abundant phyla, Bacteroidetes proportion was lower (*P* = 0.002) in the CG compared to the control and UM146 treatments, but it was not different from CGUM146 (Figure 3B, Supplementary Table 1). Classification of OTUs at lower taxonomical levels resulted in identification of 177 taxa, of which 76 were ≥ 0.01% of the community, and the rest were < 0.01% of the community. Bacterial taxa with a relative abundance of ≥0.01% of the community were analyzed using PLS-DA to identify bacteria that were most characteristic of different treatment groups and the results are shown in Figures 5A,B. Supplementary Table 3 shows a summary of mean abundances of all the taxa.

#### Ascending colon

**Figure 6:**
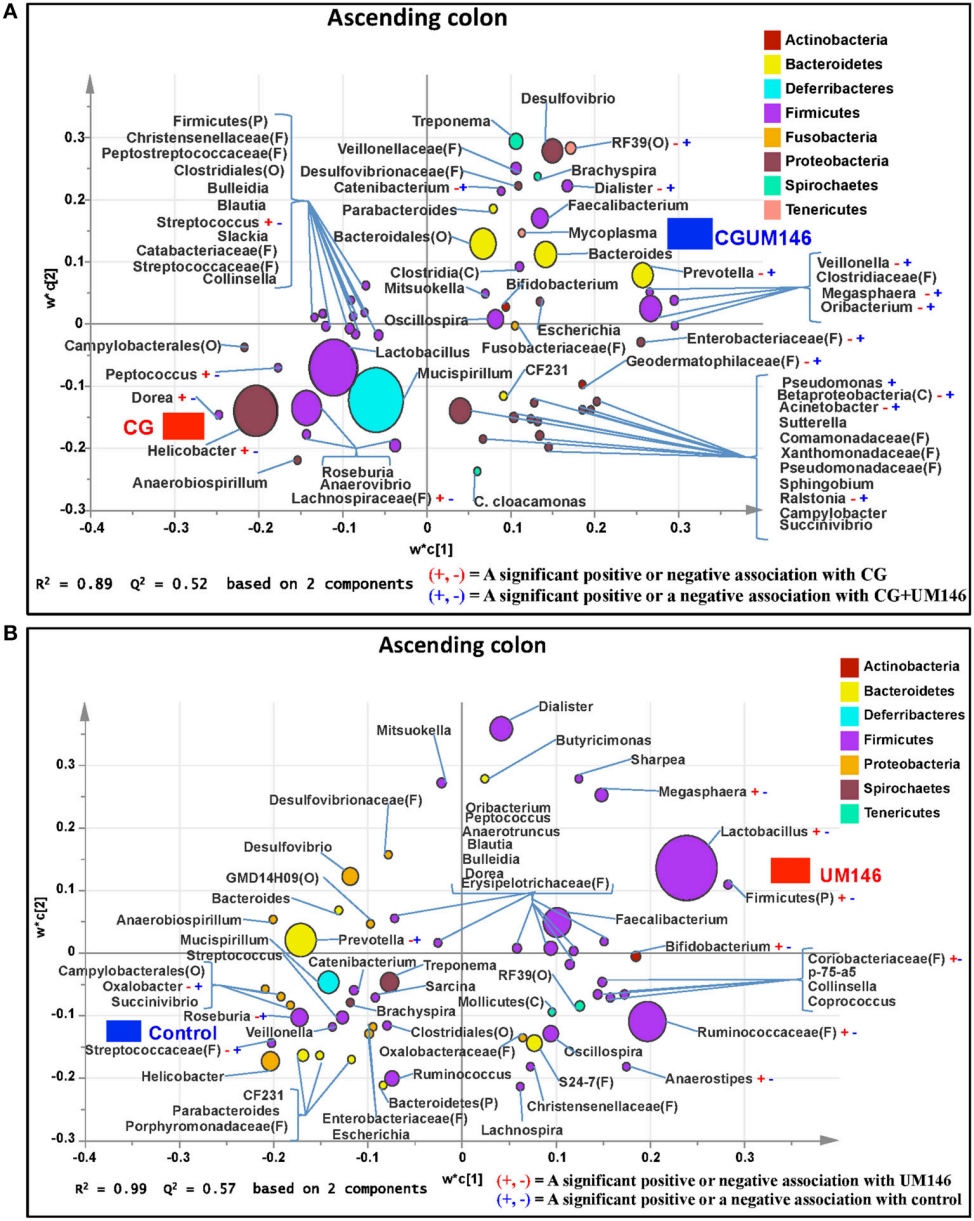
**Taxa that were positively or negatively associated with: (A) CG or CGUM146 in the ascending colon of pigs treated with CG and inoculated with adherent invasive *Escherichia coli* (AIEC) strain UM146; (B) control or UM146 in the ascending colon of pigs inoculated with AIEC UM146**. CG: pigs received 1% CG only in the drinking water daily; CGUM146: pigs received CG from day 1 and were inoculated with AIEC UM146 on day 8; UM146: pigs were inoculated with AIEC UM146 on day 8. Pigs in designated groups received 100 mL of AIEC UM146 culture (10^8^ CFU/mL) in feed.

A total of 1,293,477 quality-filtered sequences were obtained from the samples with an average of 41,725 sequences per sample. Twenty phyla were identified in all the samples. Among the most abundant phyla, the proportion of Firmicutes was lower (*P* = 0.007) in CG and CGUM146 groups, while Proteobacteria (*P* = 0.001) and Deferribacteres (*P* = 0.039) were higher in these groups compared to control and UM146 treatments. In addition, Bacteroidetes abundance was lower (*P* < 0.0001) in the CG group but not different from UM146 compared to the other treatments (Figure 3C, Supplementary Table 1). Classification of OTUs at lower taxonomical levels resulted in the identification of 232 taxa of which 81 were ≥0.01% of the community, while 151 were < 0.01% of the community. Bacterial taxa with a relative abundance of ≥0.01% of the community were analyzed using PLS-DA to identify bacteria that were most characteristic of different treatment groups. The results are shown in Figures 6A,B. Supplementary Table 4 shows a summary of mean abundances of all the taxa.

#### Descending colon

A total of 1,148,413 quality-filtered sequences were obtained from the samples with a mean of 39,600 sequences per sample. Seventeen phyla were identified in all the samples. Among the most abundant phyla, the proportion of Firmicutes and Tenericutes were lower (*P* = 0.0055 and 0.0047, respectively) in the CG and CGUM146 treatments compared to the control, but wasn't different compared to UM146. Proteobacteria abundance was higher (*P* < 0.0001) in CG treatment compared to other treatments. Conversely, the proportion of Bacteroidetes was lower (*P* < 0.0001) in CG treatment compared to other treatment groups (Figure 3D, Supplementary Table 1).

**Figure 7:**
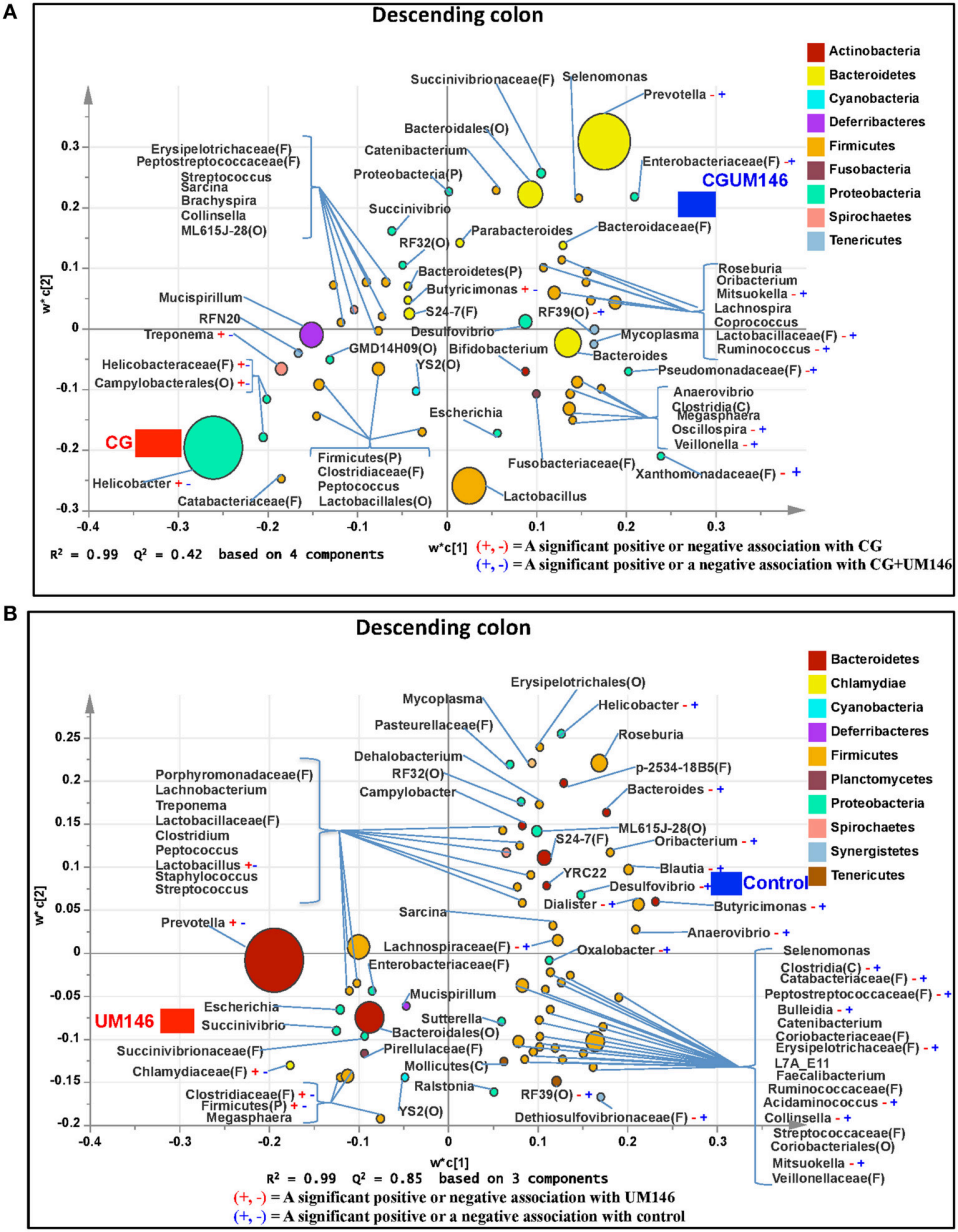
**Taxa that were positively or negatively associated with: (A) CG or CGUM146 in the descending colon of pigs treated with CG and inoculated with adherent invasive *Escherichia coli* (AIEC) strain UM146; (B) control or UM146 in the descending colon of pigs inoculated with AIEC UM146**. CG: pigs received 1% CG only in the drinking water daily; CGUM146: pigs received CG from day 1 and were inoculated with AIEC UM146 on day 8; UM146: pigs were inoculated with AIEC UM146 on day 8. Pigs in designated groups received 100 mL of AIEC UM146 culture (10^8^ CFU/mL) in feed.

Classification of OTUs at lower taxonomical levels resulted in the identification of 181 taxa, of which 84 were ≥0.01% of the community, while 97 were < 0.01% of the community. Bacterial taxa with a relative abundance of ≥0.01% of the community were analyzed using PLS-DA to identify bacteria that were most characteristic of different treatment groups. The results are shown in Figures 7A,B. Supplementary Table 5 shows a summary of mean abundance of all the taxa.

### Functional metagenome of microbiomes

A closed-reference based OUT picking step was employed for the PICRUST (v. 1.0.0-dev) analysis using the Greengenes (v.13.5) database. The proportion of reads that mapped to reference during OTU picking was 96.7% for the ileum, 91.4% for the cecum, 88.1% for the ascending colon, and 85.7% for the descending colon (an average of 90.5% for all the tissues). The data was normalized by copy numbers before metagenomes prediction, and the Nearest Sequenced Taxon Index (NSTI) for each sample which reflects the availability of reference genomes that are closely related to the abundant microorganisms in the samples were determined during metagenome prediction. On average, the NSTIs were: 0.06 for the ileum, 0.06 for the cecum, 0.10 for ascending colon, and 0.10 for descending colon. High NSTI scores (>0.15) are indicative that fewer related references are available and predictions were of low quality whereas low scores (< 0.06) are reflective of availability of closely related reference genomes.

**Figure 8:**
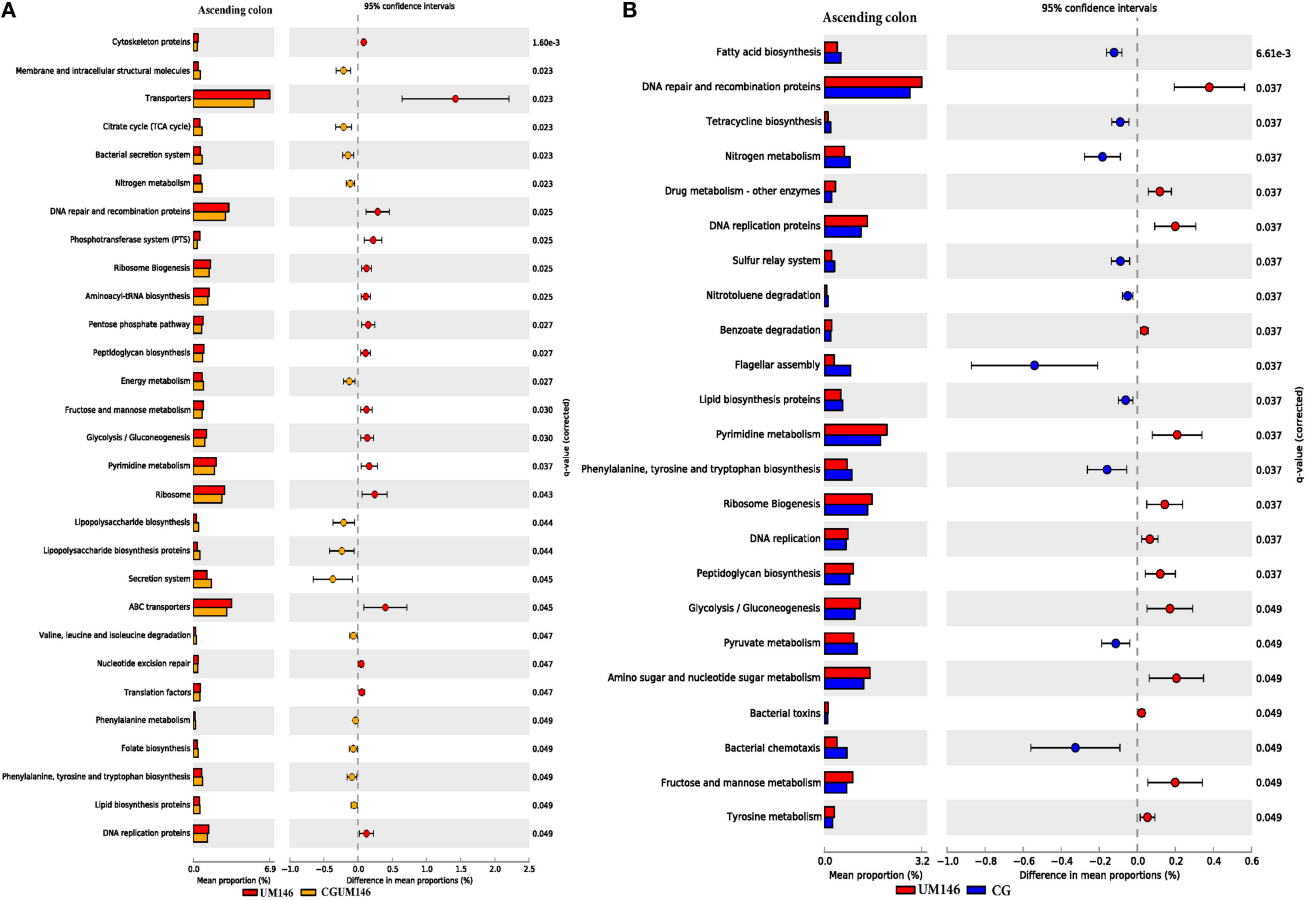
**Subsystems and pathways enriched or decreased in: (A) UM146 vs. CG; and (B) UM146 vs. CGUM146 of the ascending colon samples from pigs treated with CG or inoculated with adherent invasive *Escherichia coli* (AIEC) strain UM146**. Corrected *P*-values were calculated using the Storey's FDR correction. Subsystems or pathways overrepresented in the UM146 or (CG) samples had a positive or (negative) differences between mean proportions and were indicated by red and (blue) color, respectively. Subsystems or pathways overrepresented in the UM146 or (CGUM146) samples had a positive or (negative) difference between mean proportions and were indicated by red and (orange) color, respectively. CG: pigs received 1% CG only in the drinking water daily; CGUM146: pigs received CG from day 1 and were inoculated with AIEC UM146 on day 8; UM146: pigs were inoculated with AIEC UM146 on day 8. Pigs in designated groups received 100 mL of AIEC UM146 culture (10^8^ CFU/mL) in feed.

**Figure 9:**
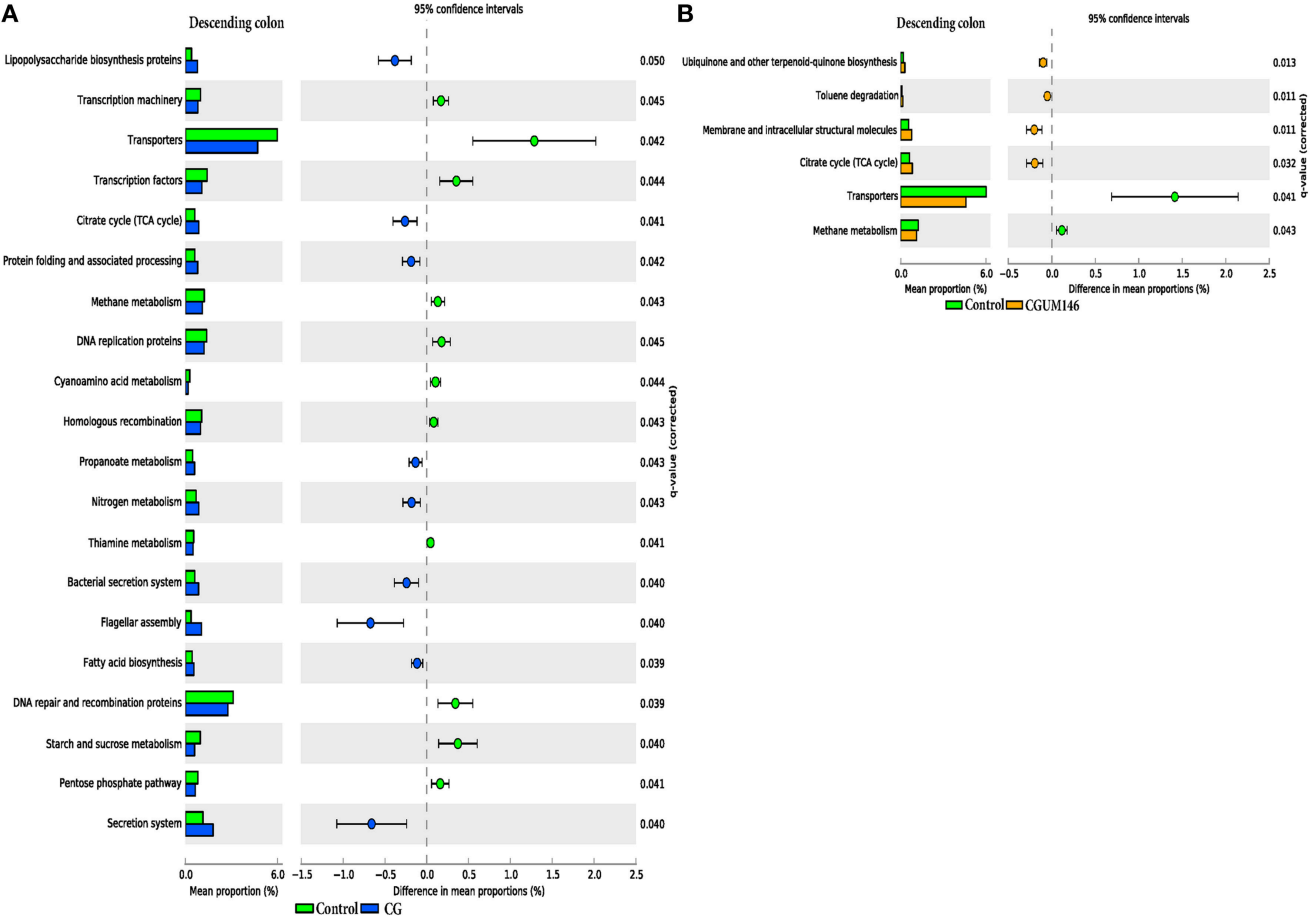
**Subsystems and pathways enriched or decreased in: (A) control vs. CG; and (B) control vs. CGUM146 of the descending colon samples from pigs treated with CG and inoculated with adherent invasive *Escherichia coli* (AIEC) strain UM146**. Corrected *P*-values were calculated using the Storey's FDR correction. Subsystems or pathways overrepresented in the control or (CG) pig descending colon samples had a positive or a (negative) difference between mean proportions and were indicated by light green and (blue) color, respectively. Subsystems or pathways overrepresented in the control or (CGUM146) pig samples had a positive or a (negative) difference between mean proportions and were indicated by light green and (orange) color, respectively. CG: pigs received 1% CG only in the drinking water daily; CGUM146: pigs received CG from day 1 and were inoculated with AIEC UM146 on day 8; UM146: pigs were inoculated with AIEC UM146 on day 8. Pigs in designated groups received 100 mL of AIEC UM146 culture (10^8^ CFU/mL) in feed.

As shown in Figure 8, different metabolic pathways were enriched in the mucosal microbiota of the ascending colon tissue in UM146 including fructose and mannose metabolism, amino sugar, and nucleotide sugar metabolism, ribosome biogenesis, DNA replication proteins, and DNA repair and recombination proteins, among others. Functional pathways enriched in the ascending colon mucosal microbiota of CG- or CGUM146-treated pigs include, but are not limited to, bacterial chemotaxis, flangellar assembly, and lipopolysaccharide biosynthesis proteins. As shown in Figure 9, different functional pathways were enriched in the mucosal microbiota of the descending colon tissue in the control group including, but not limited to, transporters, transcription factors, DNA repair, and recombination proteins, and starch and sucrose metabolism. In addition, several functional pathways were also enriched in the descending colon mucosal microbiota of CG- or CGUM146-treated pigs including secretion system, flagellar assembly, bacterial secretion system, and lipopolysaccharide biosynthesis proteins. Comparisons with the control or UM146 in the ascending and descending colon, respectively, were not significant after correcting the *P*-values and therefore, they were not included. Also, no comparisons were included for the ileum and the cecum as there were no significant differences in pathways after correcting for the *P*-values.

## Discussion

Changes in microbial composition and its interaction with the immune system play an important role in the pathogenesis of IBD (Abraham and Medzhitov, 2011; Khor et al., 2011; Manichanh et al., 2012; Sartor and Mazmanian, 2012; Hansen, 2015). In this study, we used 16S rRNA gene Illumina sequencing to investigate differences in the composition and function of mucosa-associated microbial communities of different GI tract segments in a piglet model of experimental IBD. In agreement with previous studies in IBD patients (Sokol et al., 2008; Man et al., 2011; Walters et al., 2014; Wright et al., 2015), our results showed that the relative abundance of Proteobacteria was higher (*P* = 0.001) in pigs with CG-induced colitis whilst Firmicutes and Bacteroides were lower (*P* < 0.001) compared to control in the large intestine (Figure 3, Supplementary Table 1). Also, pigs with CG-induced colitis clustered separately from the control and UM146 in the cecum and descending colon (Figure 2) indicating a distinct shift in bacterial composition, which is in line with previous observations in microbiota profiles of patients with IBD (Ott et al., 2004; Sokol et al., 2008). Metabolic capacity of the microbiome in the colon of colitic pigs was also altered (Figures 8, 9) but no significant changes were observed in UM146 treatment alone compared to the control, and neither did the combination of CG and UM146 further impact the extent of observed changes. This indicates that either *E. coli* strain UM146 did not successfully colonize the GI tract and therefore did not have much impact on microbial equilibrium and inflammation, or that its effects were transient. In addition, by combining CG-induced colitis and UM146 inoculation, it was expected that the pigs would experience adverse effects and manifest more dysbiosis and inflammation compared to the pigs with colitis alone; however, this was not the case in our study as the observed changes could only be mainly associated with the colitis and not UM146 inoculation. The lack of clear effects of UM146 could be due to the one time inoculation that was used in our study and the length of our study after inoculation. However, AIEC UM146 is well-adapted to colonize human intestines rather than pigs and, therefore, a one-time inoculation may not be sufficient for AIEC UM146 to effectively overrun a plethora of indigenous microbes that colonize pig's digestive tract. Repeated dosing over a relatively longer period of time may, therefore, provide a better insight on the cause or consequence role of this AIEC strain in experimental colitis/IBD.

The impact of experimental treatments on the microbiota of the ileum was minor compared to other tested intestinal segments. This suggests that CG-induced colitis has less effect on the ileum, which indicates that CG may exhibit different impacts on mucosal microbiota in different intestinal segments. One point worth noting is that the proportion of Bacteroidetes was consistently lower in the large intestine of pigs with CG-induced colitis compared to the colitic pigs that were also inoculated with UM146 (Figure 3, Supplementary Table 1). This might suggest that UM146 induced or enhanced proliferation of Bacteroidetes in pigs with colitis, which is in agreement with some previous studies in patients with CD that have reported increase in Bacteroidetes, based on a recent systematic review (Wright et al., 2015). The proliferation of Proteobacteria, especially AIEC, has been consistently reported in IBD patients and in experimental models of IBD, but it is still not clear if members of this phylum cause inflammation or if their proliferation is a result of inflammatory status in the gut (Mukhopadhya et al., 2012). A recent study (Chassaing et al., 2014) found that transient colonization with AIEC is sufficient to trigger chronic intestinal inflammation in genetically susceptible hosts–mice lacking Toll-like receptor 5 (TLR5), the sensor for bacterial flagellin. Such chronic inflammation lasted even when AIEC was no longer detectable and was also accompanied by alterations in fecal microbial composition, loss of species diversity and higher levels of LPS and flagellin. None of these alterations were observed in wild-type mice/non-susceptible healthy hosts. This supports a causal role of AIEC in IBD in genetically susceptible hosts, but a consequent role in non-susceptible hosts, and could partially explain our results in which AIEC strain UM146 alone did not have much effect on inflammation or microbial dysbiosis. However, because intestinal inflammation may still persist even after AIEC is undetectable in the susceptible host, AIEC may be an intestinal arsonist pathobiont that ignites IBD and flees the scene in genetically susceptible hosts (Jellbauer and Raffatellu, 2014). Still, it is not clear whether the same process happens in healthy hosts that are not genetically susceptible to IBD, which could also be the reason why the pigs in our study did not have diarrhea beyond 48 h after inoculation.

We identified bacterial taxa that were consistently associated with particular treatments in all the four intestinal segments examined, or shared in between two or three intestinal sections (Figures 4–7). For example, *Lactobacillus* and *Megasphaera* were positively associated with UM146 while *Lactobacillus, Helicobacter*, and unclassified members of Streptococcaceae were positively associated with CG treatment. On the other hand, *Prevotella, Ruminococcus, Oribacterium, Escherichia, Coprococcus, Bulleidia, Veillonella, Mitsuokella*, and unclassified members of Enterobacteriaceae and RF39 were positively associated with CGUM146. The mechanism by which UM146 and CG treatments were associated with *Lactobacillus* growth is difficult to elucidate. It could be speculated that the changes induced by the presence of CG or UM146 may favor specific bacterial colonization. Moreover, the increased presence of *Lactobacillus* in pigs exposed to UM146 and CG could be relevant since these bacteria are protective against infections including inflammatory and infectious diseases (Lara-Villoslada et al., 2007). *Lactobacillus* may also produce lactate that can be utilized by other bacteria to produce short chain fatty acids (SCFA), which are metabolites that are well-known for their health benefits (Tsukahara et al., 2002), although our correlation analysis did not reveal a strong association between some of these taxa and various SCFA. *Helicobacter pylori* is commonly found in the gut and under normal gut conditions, it does not cause adverse effects; however, it is a major reason for the genesis of gastritis and peptic ulcers in humans. Colonization of gnotobiotic piglets with *H. pylori* is also associated with gastritis and gastric ulcers (Heinritz et al., 2013). Interestingly, *H. pylori* has been found to be negatively correlated with IBD, which may be explained by the “hygiene hypothesis”, and only non-*pylori Helicobacters* including *Helicobacter hepaticus* have been shown to induce colitis in rodent models of experimental colitis (Hold et al., 2014). It is therefore reassuring that perhaps *H. hepaticus* was the major species associated with the CG treatment in the colon in our study, even though our data was only classified to the genus level and therefore it was not possible to definitively discriminate between different species of the g. *Helicobacter*.

Some of the taxa were shared/positively associated with specific treatments across different intestinal compartments, which may imply strong relations between the taxa and treatment conditions. Some taxa were positively correlated with certain inflammatory cytokines/SCFAs (Table 4, Supplementary Figures 1–3), reinforcing that different taxa exhibit redundancy and pleiotropy, which makes elucidation on their association with specific treatments difficult. However, increased levels of Enterobacteriaceae have been repeatedly reported in IBD patients, especially in CD, although results related, but not limited, to Enterobacteriaceae, *E. coli, Bacteroides*, and *Lactobacillus* species are not consistent between studies (Takaishi et al., 2008; Andoh et al., 2011; Wright et al., 2015). Such differences may be explained by variations in sample sources, sampling locations, analytical methodologies and disease activity among other confounding factors in separate studies. Moreover, we only analyzed samples collected at one time point, which is a limitation of our study and possibly many other studies as it is not possible to discriminate between “normal” changes and pathological conditions, and between microbes that are transient and those that are permanent residents of the gut.

**Table 4:** **Correlation coefficient between selected taxa^a^ and short chain fatty acids (acetic, propionic, butyric acid) and inflammatory markers in the ileum, cecum and ascending colon**.

**GI segment**	**Taxa**	**Cytokine or SCFA**	**Rho (ρ)**	***P*-values**
Ileum	g. *Ruminococcus*	IL-1β	0.4714	0.026
		IL-6	0.4987	0.018
		IL-10	0.4498	0.035
	f. Enterobacteriaceae	IL-1β	0.4254	0.048
		IL-8	0.5594	0.006
	g. *Prevotella*	IL-6	0.4411	0.039
		IL-10	0.4509	0.035
	f. Clostridiaceae	IL-6	0.6862	< 0.001
	g. *Escherichia*	IL-8	0.4226	0.050
	g. *Faecalibacterium*	IL-10	0.5439	0.008
		Butyrate	0.4696	0.027
	g. *Lactobacillus*	IL-10	−0.4617	0.030
	g. *Helicobacter*	Acetate	0.4229	0.049
	g. *Streptococcus*	Propionate	0.4336	0.043
Cecum	g. *Megasphaera*	IL-1β	0.4743	0.022
	g. *Desulfovibrio*	IL-10	0.58	0.003
		TNF-α	0.4599	0.027
	o. Bacteroidales	Acetate	0.4555	0.028
	g. *Mucispirillum*	Acetate	−0.579	0.003
		Proprionate	−0.4328	0.039
		Butyrate	−0.499	0.015
	f. Erysipelotrichaceae	Acetate	−0.5365	0.008
		Butyrate	−0.5622	0.005
	g. *Anaerovibrio*	Proprionate	−0.4367	0.037
	g. *Prevotella*	Butyrate	0.4555	0.028
	g. *Roseburia*	Butyrate	−0.4189	0.046
	g. *Oscillospira*	Butyrate	−0.4555	0.028
Ascending colon	o. Clostridiales	IL-6	0.5474	0.005
		IL-8	−0.54	0.006
		Acetate	0.6139	0.001
	f. Ruminococcaceae	IL-6	0.5062	0.011
		IL-8	−0.4426	0.030
		Acetate	0.6573	< 0.001
	g. *Oscillospira*	IL-6	0.5092	0.011
		IL-8	−0.4773	0.018
		Acetate	0.5391	0.006
	g. *Ruminococcus*	IL-6	0.5728	0.003
		IL-8	−0.4547	0.025
		Acetate	0.5426	0.006
		Butyrate	0.4539	0.025
	g. *Desulfovibrio*	IL-6	0.6121	0.001
		IL-8	0.6121	0.001
		Acetate	−0.4287	0.036
		Butyrate	−0.5887	0.002
	g. *Mucispirillum*	IL-6	−0.4274	0.037
		IL-8	0.5634	0.004
		Acetate	−0.7356	< 0.001
		Butyrate	−0.6173	0.001
	o. RF39	IL-6	−0.4134	0.044
		IL-8	−0.4134	0.044
		Acetate	0.4569	0.024
	g. *Bacteroides*	IL-8	0.5649	0.004
		Acetate	−0.5231	0.008
	g. *Prevotella*	IL-8	−0.433	0.034
	f. S24-7	IL-6	0.4835	0.016
		IL-8	−0.4791	0.017
		IL-10	−0.414	0.044
		Acetate	0.7452	< 0.001
	g. *CF231*	IL-8	−0.4596	0.023
	g. *Helicobacter*	IL-10	−0.4066	0.048
	f. Veillonellaceae	TNF-α	−0.5579	0.004
	g. *Blautia*	Acetate	0.4321	0.034
	g. *Treponema*	Propionate	0.4217	0.040
	g. *Lactobacillus*	Propionate	−0.4939	0.014
	f. Streptococcaceae	Propionate	−0.452	0.026
		Butyrate	−0.4295	0.036
	g. *Campylobacter*	Acetate	−0.5188	0.009
		Butyrate	−0.5366	0.006
	g. *Campylobacter*	Acetate	−0.5188	0.009
		Butyrate	−0.5366	0.006

a Taxa with relative abundance ≥0.5% of the community were used for the correlation analysis and only the significant correlations are shown in the Table. SCFA, short chain fatty acid.

Microbial activities in the gastrointestinal tract can result in production of different metabolites, such as SCFA (for example butyrate), substances with known immunoregulatory properties that greatly contribute to gut health (Sartor, 2008; Canani et al., 2011; Smith et al., 2013). We observed a lower level of butyrate in the cecum and colon digesta of pigs receiving CG (Table 2), a condition that may be associated with poor colonocyte development and an increase in inflammatory markers. However, we did not observe much elevation in the inflammatory markers in CG-treated pigs although the analysis was only performed once and at the end of the experiment, and therefore, some changes in cytokine levels during the course of the study may have been missed. Since perturbations of the gut microbiota that lead to pathological conditions are still not fully understood, determining what aspects of the gut microbiota structurally and functionally change in IBD conditions remains an important part of research. Based on our functional metagenome prediction results (Figures 8, 9), most of the categories associated with the mucosal microbiota in control pigs that did not differ from the pigs in the UM146 treatment included sugars, starch, and sucrose metabolism, DNA replication, repair, and recombination proteins, and transcription factors. Conversely, the large intestine's microbiota in CG-induced colitis (both CG and CGUM146) was enriched with capacities associated with secretion systems, lipopolysaccharide biosynthesis proteins, membrane and structural molecules, and flagellar assembly. Therefore, gut microbiota that were associated with CG-induced colitis appear to have a reduced capacity for energy acquisition and a dysregulated microbial signaling and repair pathways. Lipopolysaccharide bacterial structures are known drivers of inflammation whilst flagellar bacterial antigens have been implicated as disease drivers in both mice models of colitis and in IBD patients (Steiner, 2007).

In conclusion, our study has demonstrated that induction of colitis using 1% CG caused intestinal bacterial dysbiosis. Certain bacterial shifts observed in the distal GI tract as a result of CG treatment are consistent with previous findings in IBD patients. In this context, CG significantly influenced bacterial diversity and induced notable microbial changes in the percentages of the major phyla Firmicutes, Bacteroidetes, and Proteobacteria, especially in the cecum, ascending, and descending colon. No significant difference was observed between UM146 and the control group, suggesting that the ability of UM146 to cause bacterial dysbiosis may be limited in healthy subjects—a known characteristic of opportunistic pathogens. Overall, CG was shown to be an acceptable model for mimicking human colitis in pigs, but the role of UM146 in IBD needs further investigation.

## Author contributions

EK and JEG conceived and designed the study. PMM conducted the experiment and performed the analyses. EK, PMM, JEG, and SS interpreted the data and wrote the manuscript.

## Funding

The research is funded by Start Up grants from University of Manitoba.

### Conflict of interest statement

The authors declare that the research was conducted in the absence of any commercial or financial relationships that could be construed as a potential conflict of interest.

## Supplementary material

Supplementary Figure 1 **Correlation coefficient of selected taxa with the short chain fatty acids (acetic, propionic and butyric acid) and inflammatory markers in the ileum**. The blue color shows a positive correlation while the coral color shows a negative correlation. The intensity of the color depicts the strength of the correlation.

Supplementary Figure 2 **Correlation coefficient of selected taxa with the short chain fatty acids (acetic, propionic and butyric acid) and inflammatory markers in the cecum**. The blue color shows a positive correlation while the coral color shows a negative correlation. The intensity of the color depicts the strength of the correlation.

Supplementary Figure 3 **Correlation coefficient of selected taxa with the short chain fatty acids (acetic, propionic and butyric acid) and inflammatory markers in the descending colon**. The blue color shows a positive correlation while the coral color shows a negative correlation. The intensity of the color depicts the strength of the correlation.

Supplementary Table 1 **Analysis of the most abundant phyla in each intestinal segment of pigs treated with carrageenan gum (CG)^1^ and inoculated with adhered invasive *Escherichia coli* (AIEC) strain UM146^2^**.

Supplementary Table 2 **A summary showing mean relative abundances of taxa in ileal mucosa samples**.

Supplementary Table 3 **A summary showing mean relative abundances of taxa in cecal mucosa samples**.

Supplementary Table 4 **A summary showing mean relative abundances of taxa in the ascending colon mucosa samples**.

Supplementary Table 5 **A summary showing mean relative abundances of taxa in the descending colon mucosa samples**.

## References

[B1] Abraham C. Medzhitov R. (2011). Interactions between the host innate immune system and microbes in inflammatory bowel disease. Gastroenterology 140, 1729–1737. 10.1053/j.gastro.2011.02.012 21530739 PMC4007055

[B2] Agus A. Massier S. Darfeuille-Michaud A. Billard E. Barnich N. (2014). Understanding host-adherent-invasive *Escherichia coli* interaction in Crohn's disease: opening up new therapeutic strategies. Biomed. Res. Int. 2014:567929. 10.1155/2014/567929 25580435 PMC4279263

[B3] Anderson M. (2005). PERMANOVA: A FORTRAN Computer Program For Permutational Multivariate Analysis Of Variance, 24th Edn. Department of Statistics, University of Auckland.

[B4] Andoh A. Imaeda H. Aomatsu T. Inatomi O. Bamba S. Sasaki M.. (2011). Comparison of the fecal microbiota profiles between ulcerative colitis and Crohn's disease using terminal restriction fragment length polymorphism analysis. J. Gastroenterol. 46, 479–486. 10.1007/s00535-010-0368-4 21253779

[B5] Bhandari S. K. Ominski K. H. Wittenberg K. M. Plaizier J. C. (2007). Effects of chop length of alfalfa and corn silage on milk production and rumen fermentation of dairy cows. J. Dairy Sci. 90, 2355–2366. 10.3168/jds.2006-609 17430939

[B6] CCAC (1993). Guide to the Care and Use of Experimental Animal. Ottawa, ON: Canadian Council of Animal Care.

[B7] Canani R. B. Costanzo M. D. Leone L. Pedata M. Meli R. Calignano A. (2011). Potential beneficial effects of butyrate in intestinal and extraintestinal diseases. World J. Gastroenterol. 17, 1519–1528. 10.3748/wjg.v17.i12.1519 21472114 PMC3070119

[B8] Cao Y. Shen J. Ran Z. H. (2014). Association between *Faecalibacterium prausnitzii* reduction and inflammatory bowel disease: a meta-analysis and systematic review of the literature. Gastroenterol. Res. Pract. 2014:872725. 10.1155/2014/872725 24799893 PMC3985188

[B9] Caporaso J. G. Bittinger K. Bushman F. D. DeSantis T. Z. Andersen G. L. Knight R. (2010a). PyNAST: a flexible tool for aligning sequences to a template alignment. Bioinformatics 26, 266–267. 10.1093/bioinformatics/btp636 19914921 PMC2804299

[B10] Caporaso J. G. Kuczynski J. Stombaugh J. Bittinger K. Bushman F. D. Costello E. K.. (2010b). QIIME allows analysis of high-throughput community sequencing data. Nat. Methods 7, 335–336. 10.1038/nmeth.f.303 20383131 PMC3156573

[B11] Caporaso J. G. Lauber C. L. Walters W. A. Berg-Lyons D. Huntley J. Fierer N.. (2012). Ultra-high-throughput microbial community analysis on the Illumina HiSeq and MiSeq platforms. ISME J. 6, 1621–1624. 10.1038/ismej.2012.8 22402401 PMC3400413

[B12] Chapman R. W. Selby W. S. Jewell D. P. (1986). Controlled trial of intravenous metronidazole as an adjunct to corticosteroids in severe ulcerative colitis. Gut 27, 1210–1212. 10.1136/gut.27.10.1210 3536677 PMC1433885

[B13] Chassaing B. Koren O. Carvalho F. A. Ley R. E. Gewirtz A. T. (2014). AIEC pathobiont instigates chronic colitis in susceptible hosts by altering microbiota composition. Gut 63, 1069–1080. 10.1136/gutjnl-2013-304909 23896971 PMC4089387

[B14] Darfeuille-Michaud A. Boudeau J. Bulois P. Neut C. Glasser A. L. Barnich N.. (2004). High prevalence of adherent-invasive *Escherichia coli* associated with ileal mucosa in Crohn's disease. Gastroenterology 127, 412–421. 10.1053/j.gastro.2004.04.061 15300573

[B15] Darfeuille-Michaud A. Neut C. Barnich N. Lederman E. Di Martino P. Desreumaux P.. (1998). Presence of adherent *Escherichia coli* strains in ileal mucosa of patients with Crohn's disease. Gastroenterology 115, 1405–1413. 10.1016/S0016-5085(98)70019-8 9834268

[B16] Derakhshani H. Tun H. M. Khafipour E. (2016). An extended single-index multiplexed 16S rRNA sequencing for microbial community analysis on MiSeq Illumina platforms. J. Basic Microbiol. 56, 321–326. 10.1002/jobm.201500420 26426811

[B17] DeSantis T. Z. Hugenholtz P. Larsen N. Rojas M. Brodie E. L. Keller K.. (2006). Greengenes, a chimera-checked 16S rRNA gene database and workbench compatible with ARB. Appl. Environ. Microbiol. 72, 5069–5072. 10.1128/AEM.03006-05 16820507 PMC1489311

[B18] Desilets M. Deng X. Rao C. Ensminger A. W. Krause D. O. Sherman P. M.. (2016). Genome-based definition of an inflammatory bowel disease-associated adherent-invasive *Escherichia coli* pathovar. Inflamm. Bowel Dis. 22, 1–12. 10.1097/MIB.0000000000000574 26444104

[B19] Edgar R. C. (2010). Search and clustering orders of magnitude faster than BLAST. Bioinformatics 26, 2460–2461. 10.1093/bioinformatics/btq461 20709691

[B20] Edgar R. C. Haas B. J. Clemente J. C. Quince C. Knight R. (2011). UCHIME improves sensitivity and speed of chimera detection. Bioinformatics 27, 2194–2200. 10.1093/bioinformatics/btr381 21700674 PMC3150044

[B21] Elson C. O. Sartor R. B. Tennyson G. S. Riddell R. H. (1995). Experimental-models of inflammatory bowel-disease. Gastroenterology 109, 1344–1367. 10.1016/0016-5085(95)90599-5 7557106

[B22] Fite A. Macfarlane S. Furrie E. Bahrami B. Cummings J. H. Steinke D. T.. (2013). Longitudinal analyses of gut mucosal microbiotas in ulcerative colitis in relation to patient age and disease severity and duration. J. Clin. Microbiol. 51, 849–856. 10.1128/JCM.02574-12 23269735 PMC3592070

[B23] Frank D. N. St. Amand A. L. Feldman R. A. Boedeker E. C. Harpaz N. Pace N. R. (2007). Molecular-phylogenetic characterization of microbial community imbalances in human inflammatory bowel diseases. Proc. Natl. Acad. Sci. U.S.A. 104, 13780–13785. 10.1073/pnas.0706625104 17699621 PMC1959459

[B24] Friswell M. Campbell B. Rhodes J. (2010). The role of bacteria in the pathogenesis of inflammatory bowel disease. Gut Liver 4, 295–306. 10.5009/gnl.2010.4.3.295 20981205 PMC2956340

[B25] Guarner F. (2005). The intestinal flora in inflammatory bowel disease: normal or abnormal? Curr. Opin. Gastroenterol. 21, 414–418. 15930980

[B26] Hammer Ø. Harper D. A. T. Ryan P. D. (2001). PAST: paleontological statistics software package for education and data analysis. Palaeontol. Electron. 4, 1–9.

[B27] Hansen J. J. (2015). Immune responses to intestinal microbes in inflammatory bowel diseases. Curr. Allergy Asthma Rep. 15, 562. 10.1007/s11882-015-0562-9 26306907

[B28] Heinritz S. N. Mosenthin R. Weiss E. (2013). Use of pigs as a potential model for research into dietary modulation of the human gut microbiota. Nutr. Res. Rev. 26, 191–209. 10.1017/S0954422413000152 24134811

[B29] Hold G. L. Smith M. Grange C. Watt E. R. El-Omar E. M. Mukhopadhya I. (2014). Role of the gut microbiota in inflammatory bowel disease pathogenesis: what have we learnt in the past 10 years? World J. Gastroenterol. 20, 1192–1210. 10.3748/wjg.v20.i5.1192 24574795 PMC3921503

[B30] Honda K. Littman D. R. (2012). The microbiome in infectious disease and inflammation. Annu. Rev. Immunol. 30, 759–795. 10.1146/annurev-immunol-020711-074937 22224764 PMC4426968

[B31] Hooper L. V. Midtvedt T. Gordon J. I. (2002). How host-microbial interactions shape the nutrient environment of the mammalian intestine. Annu. Rev. Nutr. 22, 283–307. 10.1146/annurev.nutr.22.011602.092259 12055347

[B32] Horwitz B. H. (2007). The straw that stirs the drink: insight into the pathogenesis of inflammatory bowel disease revealed through the study of microflora-induced inflammation in genetically modified mice. Inflamm. Bowel Dis. 13, 490–500. 10.1002/ibd.20098 17243141

[B33] Jellbauer S. Raffatellu M. (2014). An intestinal arsonist: pathobiont ignites IBD and flees the scene. Gut 63, 1034–1035. 10.1136/gutjnl-2013-305589 24026350

[B34] Khafipour E. Li S. Plaizier J. C. Krause D. O. (2009). Rumen microbiome composition determined using two nutritional models of subacute ruminal acidosis. Appl. Environ. Microbiol. 75, 7115–7124. 10.1128/AEM.00739-09 19783747 PMC2786511

[B35] Khor B. Gardet A. Xavier R. J. (2011). Genetics and pathogenesis of inflammatory bowel disease. Nature 474, 307–317. 10.1038/nature10209 21677747 PMC3204665

[B36] Kotlowski R. Bernstein C. N. Sepehri S. Krause D. O. (2007). High prevalence of *Escherichia coli* belonging to the B2+D phylogenetic group in inflammatory bowel disease. Gut 56, 669–675. 10.1136/gut.2006.099796 17028128 PMC1942160

[B37] Krause D. O. Little A. C. Dowd S. E. Bernstein C. N. (2011). Complete genome sequence of adherent invasive *Escherichia coli* UM146 isolated from Ileal Crohn's disease biopsy tissue. J. Bacteriol. 193, 583–583. 10.1128/JB.01290-10 21075930 PMC3019814

[B38] Langille M. G. I. Zaneveld J. Caporaso J. G. McDonald D. Knights D. Reyes J. A.. (2013). Predictive functional profiling of microbial communities using 16S rRNA marker gene sequences. Nat. Biotechnol. 31, 814–821. 10.1038/nbt.2676 23975157 PMC3819121

[B39] Lara-Villoslada F. Olivares M. Sierra S. Rodríguez J. M. Boza J. Xaus J. (2007). Beneficial effects of probiotic bacteria isolated from breast milk. Br. J. Nutr. 98(Suppl. 1), S96–S100. 10.1017/S0007114507832910 17922969

[B40] Li R. Khafipour E. Krause D. O. Entz M. H. de Kievit T. R. Fernando W. G. D. (2012). Pyrosequencing reveals the influence of organic and conventional farming systems on bacterial communities. PLoS ONE 7:e51897. 10.1371/journal.pone.0051897 23284808 PMC3526490

[B41] Lupp C. Robertson M. L. Wickham M. E. Sekirov I. Champion O. L. Gaynor E. C.. (2007). Host-mediated inflammation disrupts the intestinal microbiota and promotes the overgrowth of Enterobacteriaceae. Cell Host Microbe 2, 119–129. 10.1016/j.chom.2007.06.010 18005726

[B42] Maloy K. J. Powrie F. (2011). Intestinal homeostasis and its breakdown in inflammatory bowel disease. Nature 474, 298–306. 10.1038/nature10208 21677746

[B43] Man S. M. Kaakoush N. O. Mitchell H. M. (2011). The role of bacteria and pattern-recognition receptors in Crohn's disease. Nat. Rev. Gastroenterol. Hepatol. 8, 152–168. 10.1038/nrgastro.2011.3 21304476

[B44] Manichanh C. Borruel N. Casellas F. Guarner F. (2012). The gut microbiota in IBD. Nat. Rev. Gastroenterol. Hepatol. 9, 599–608. 10.1038/nrgastro.2012.152 22907164

[B45] Manichanh C. Rigottier-Gois L. Bonnaud E. Gloux K. Pelletier E. Frangeul L.. (2006). Reduced diversity of faecal microbiota in Crohn's disease revealed by a metagenomic approach. Gut 55, 205–211. 10.1136/gut.2005.073817 16188921 PMC1856500

[B46] Marcus S. N. Marcus A. J. Marcus R. Ewen S. W. Watt J. (1992). The pre-ulcerative phase of carrageenan-induced colonic ulceration in the guinea-pig. Int. J. Exp. Pathol. 73, 515–526. 1356411 PMC2002361

[B47] Marquardt R. R. Jin L. Z. Kim J. W. Fang L. Frohlich A. A. Baidoo S. K. (1999). Passive protective effect of egg-yolk antibodies against enterotoxigenic *Escherichia coli* K88+ infection in neonatal and early-weaned piglets. FEMS Immunol. Med. Microbiol. 23, 283–288. 10.1111/j.1574-695X.1999.tb01249.x 10225287

[B48] Martin H. M. Campbell B. J. Hart C. A. Mpofu C. Nayar M. Singh R.. (2004). Enhanced *Escherichia coli* adherence and invasion in Crohn's disease and colon cancer. Gastroenterology 127, 80–93. 10.1053/j.gastro.2004.03.054 15236175

[B49] Masella A. P. Bartram A. K. Truszkowski J. M. Brown D. G. Neufeld J. D. (2012). PANDAseq: PAired-eND Assembler for Illumina sequences. BMC Bioinformatics 13:31. 10.1186/1471-2105-13-31 22333067 PMC3471323

[B50] Miller E. R. Ullrey D. E. (1987). The pig as a model for human-nutrition. Annu. Rev. Nutr. 7, 361–382. 10.1146/annurev.nu.07.070187.002045 3300739

[B51] Miquel S. Peyretaillade E. Claret L. de Vallée A. Dossat C. Vacherie B.. (2010). Complete genome sequence of Crohn's Disease-associated adherent-invasive *E. coli* strain LF82. PLoS ONE 5:e12714. 10.1371/journal.pone.0012714 20862302 PMC2941450

[B52] Morgan X. C. Tickle T. L. Sokol H. Gevers D. Devaney K. L. Ward D. V.. (2012). Dysfunction of the intestinal microbiome in inflammatory bowel disease and treatment. Genome Biol. 13:R79. 10.1186/gb-2012-13-9-r79 23013615 PMC3506950

[B53] Mukhopadhya I. Hansen R. El-Omar E. M. Hold G. L. (2012). IBD-what role do proteobacteria play? Nat. Rev. Gastroenterol. Hepatol. 9, 219–230. 10.1038/nrgastro.2012.14 22349170

[B54] Mylonaki M. Rayment N. B. Rampton D. S. Hudspith B. N. Brostoff J. (2005). Molecular characterization of rectal mucosaassociated bacterial flora in inflammatory bowel disease. Infamm. Bowel Dis. 11, 481–487. 10.1097/01.MIB.0000159663.62651.4f 15867588

[B55] Novozamsky I. R. Eck V. Schouwenburg J. C. H. Walinga F. (1974). Total nitrogen determination in plant material by means of the indole-phenol blue method. Neth. J. Agri. Sci. 22, 3–5.

[B56] NRC (2012). Nutrient Requirements of Swine. Washington, DC: National Academies Press.

[B57] Ott S. J. Musfeldt M. Wenderoth D. F. Hampe J. Brant O. Fölsch U. R.. (2004). Reduction in diversity of the colonic mucosa associated bacterial microflora in patients with active inflammatory bowel disease. Gut 53, 685–693. 10.1136/gut.2003.025403 15082587 PMC1774050

[B58] Parks D. H. Beiko R. G. (2010). Identifying biologically relevant differences between metagenomic communities. Bioinformatics 26, 715–721. 10.1093/bioinformatics/btq041 20130030

[B59] Price M. N. Dehal P. S. Arkin A. P. (2010). FastTree 2–approximately maximum-likelihood trees for large alignments. PLoS ONE 5:e9490. 10.1371/journal.pone.0009490 20224823 PMC2835736

[B60] Qiu X. Zhang M. Yang X. Hong N. Yu C. (2013). *Faecalibacterium prausnitzii* upregulates regulatory T cells and anti-inflammatory cytokines in treating TNBS-induced colitis. J. Crohns. Colitis 7, e558–e568. 10.1016/j.crohns.2013.04.002 23643066

[B61] Rideout J. R. He Y. Navas-Molina J. A. Walters W. A. Ursell L. K. Gibbons S. M.. (2014). Subsampled open-reference clustering creates consistent, comprehensive OTU definitions and scales to billions of sequences. PeerJ 2:e545. 10.7717/peerj.545 25177538 PMC4145071

[B62] Sartor R. B. (2008). Microbial influences in inflammatory bowel diseases. Gastroenterology 134, 577–594. 10.1053/j.gastro.2007.11.059 18242222

[B63] Sartor R. B. Mazmanian S. K. (2012). Intestinal microbes in inflammatory bowel diseases. Am. J. Gastroenterol. Suppl. 1, 15–21. 10.1038/ajgsup.2012.4

[B64] Schuppler M. Lötzsch K. Waidmann M. Autenrieth I. B. (2004). An abundance of *Escherichia coli* is harbored by the mucosa- associated bacterial flora of interleukin-2-deficient mice. Infect. Immun. 72, 1983–1990. 10.1128/IAI.72.4.1983-1990.2004 15039318 PMC375167

[B65] Sellon R. K. Tonkonogy S. Schultz M. Dieleman L. A. Grenther W. Balish E.. (1998). Resident enteric bacteria are necessary for development of spontaneous colitis and immune system activation in interleukin-10-deficient mice. Infect. Immun. 66, 5224–5231. 9784526 10.1128/iai.66.11.5224-5231.1998 PMC108652

[B66] Sepehri S. Khafipour E. Bernstein C. N. Coombes B. K. Pilar A. V. Karmali M.. (2011). Characterization of *Escherichia coli* isolated from gut biopsies of newly diagnosed patients with inflammatory bowel disease. Inflamm. Bowel Dis. 17, 1451–1463. 10.1002/ibd.21509 21674703

[B67] Sepehri S. Kotlowski R. Bernstein C. N. Krause D. O. (2007). Microbial diversity of inflamed and noninflamed gut biopsy tissues in inflammatory bowel disease. Inflamm. Bowel Dis. 13, 675–683. 10.1002/ibd.20101 17262808

[B68] Sepehri S. Kotlowski R. Bernstein C. N. Krause D. O. (2009). Phylogenetic analysis of inflammatory bowel disease associated *Escherichia coli* and the fimH virulence determinant. Inflamm. Bowel Dis. 15, 1737–1745. 10.1002/ibd.20966 19462430

[B69] Smith P. M. Howitt M. R. Panikov N. Michaud M. Gallini C. A. Bohlooly-Y M.. (2013). The microbial metabolites, short-chain fatty acids, regulate colonic Treg cell homeostasis. Science 341, 569–573. 10.1126/science.1241165 23828891 PMC3807819

[B70] Sokol H. Lay C. Seksik P. Tannock G. W. (2008). Analysis of bacterial bowel communities of IBD patients: what has it revealed? Inflamm. Bowel Dis. 14, 858–867. 10.1002/ibd.20392 18275077

[B71] Sokol H. Seksik P. Furet J. P. Firmesse O. Nion-Larmurier I. Beaugerie L.. (2009). Low counts of *Faecalibacterium prausnitzii* in colitis microbiota. Inflamm. Bowel Dis. 15, 1183–1189. 10.1002/ibd.20903 19235886

[B72] Steiner T. S. (2007). How flagellin and toll-like receptor 5 contribute to enteric infection. Infect. Immun. 75, 545–552. 10.1128/IAI.01506-06 17118981 PMC1828527

[B73] Takaishi H. Matsuki T. Nakazawa A. Takada T. Kado S. Asahara T.. (2008). Imbalance in intestinal microflora constitution could be involved in the pathogenesis of inflammatory bowel disease. Int. J. Med. Microbiol. 298, 463–472. 10.1016/j.ijmm.2007.07.016 17897884

[B74] Tobacman J. (2001). Review of harmful gastrointestinal effects of carrageenan in animal experiments. Environ. Health Perspect. 109, 983–994. 10.1289/ehp.01109983 11675262 PMC1242073

[B75] Tong H. K. Lee K. H. Wong H. A. (1980). The molecular-weight and viscosity of the water-soluble polysaccharide(S) from *Eucheuma-Spinosum*. Carbohydr. Res. 81, 1–6. 10.1016/S0008-6215(00)85671-2

[B76] Tsukahara T. Koyama H. Okada M. Ushida K. (2002). Stimulation of butyrate production by gluconic acid in batch culture of pig cecal digesta and identification of butyrate-producing bacteria. J. Nutr. 132, 2229–2234. 12163667 10.1093/jn/132.8.2229

[B77] van Kruiningen H. J. (1995). On the use of antibiotics in Crohn's disease J. Clin. Gastroenterol. 20, 310–316. 10.1097/00004836-199506000-00012 7665821

[B78] Vijay-Kumar M. Aitken J. D. Carvalho F. A. Cullender T. C. Mwangi S. Srinivasan S.. (2010). Metabolic syndrome and altered gut microbiota in mice lacking Toll-like receptor 5. Science 328, 228–231. 10.1126/science.1179721 20203013 PMC4714868

[B79] Vijay-Kumar M. Sanders C. J. Taylor R. T. Kumar A. Aitken J. D. Sitaraman S. V.. (2007). Deletion of TLR5 results in spontaneous colitis in mice. J. Clin. Invest. 117, 3909–3921. 10.1172/jci33084 18008007 PMC2075480

[B80] Walters W. A. Xu Z. Knight R. (2014). Meta-analyses of human gut microbes associated with obesity and IBD. FEBS Lett. 588, 4223–4233. 10.1016/j.febslet.2014.09.039 25307765 PMC5050012

[B81] Wang Q. Garrity G. M. Tiedje J. M. Cole J. R. (2007). Naive Bayesian classifier for rapid assignment of rRNA sequences into the new bacterial taxonomy. Appl. Environ. Microbiol. 73, 5261–5267. 10.1128/AEM.00062-07 17586664 PMC1950982

[B82] Wang Y. Ames N. P. Tun H. M. Tosh S. M. Jones P. J. Khafipour E. (2016). High molecular weight barley β-glucan alters gut microbiota toward reduced cardiovascular disease risk. Front. Microbiol. 7:129. 10.3389/fmicb.2016.00129 26904005 PMC4748052

[B83] Watt J. McLean C. Marcus R. (1979). Degradation of carrageenan for the experimental production of ulcers in the colon. J. Pharm. Pharmacol. 31, 645–646. 10.1111/j.2042-7158.1979.tb13614.x 41075

[B84] Weiner M. L. (1991). Toxicological properties of carrageenan. Agents Actions 32, 46–51. 10.1007/BF01983307 2058470

[B85] Wright E. K. Kamm M. A. Teo S. M. Inouye M. Wagner J. Kirkwood C. D. (2015). Recent advances in characterizing the gastrointestinal microbiome in Crohn's disease: a systematic review. Inflamm. Bowel Dis. 21, 1219–1228. 10.1097/MIB.0000000000000382 25844959 PMC4450900

[B86] Xenoulis P. G. Palculict B. Allenspach K. Steiner J. M. Van House A. M. Suchodolski J. S. (2008). Molecular-phylogenetic characterization of microbial communities imbalances in the small intestine of dogs with inflammatory bowel disease. FEMS Microbiol. Ecol. 66, 579–589. 10.1111/j.1574-6941.2008.00556.x 18647355

