# Supplementary figures and images for: Carrageenan Gum and Adherent Invasive Escherichia coli in a Piglet Model of Inflammatory Bowel Disease: Impact on Intestinal Mucosa-associated Microbiota
Source: Front Microbiol. 2016 Apr 5;7:462. doi: 10.3389/fmicb.2016.00462 (PMC4820460; doi:10.3389/fmicb.2016.00462)

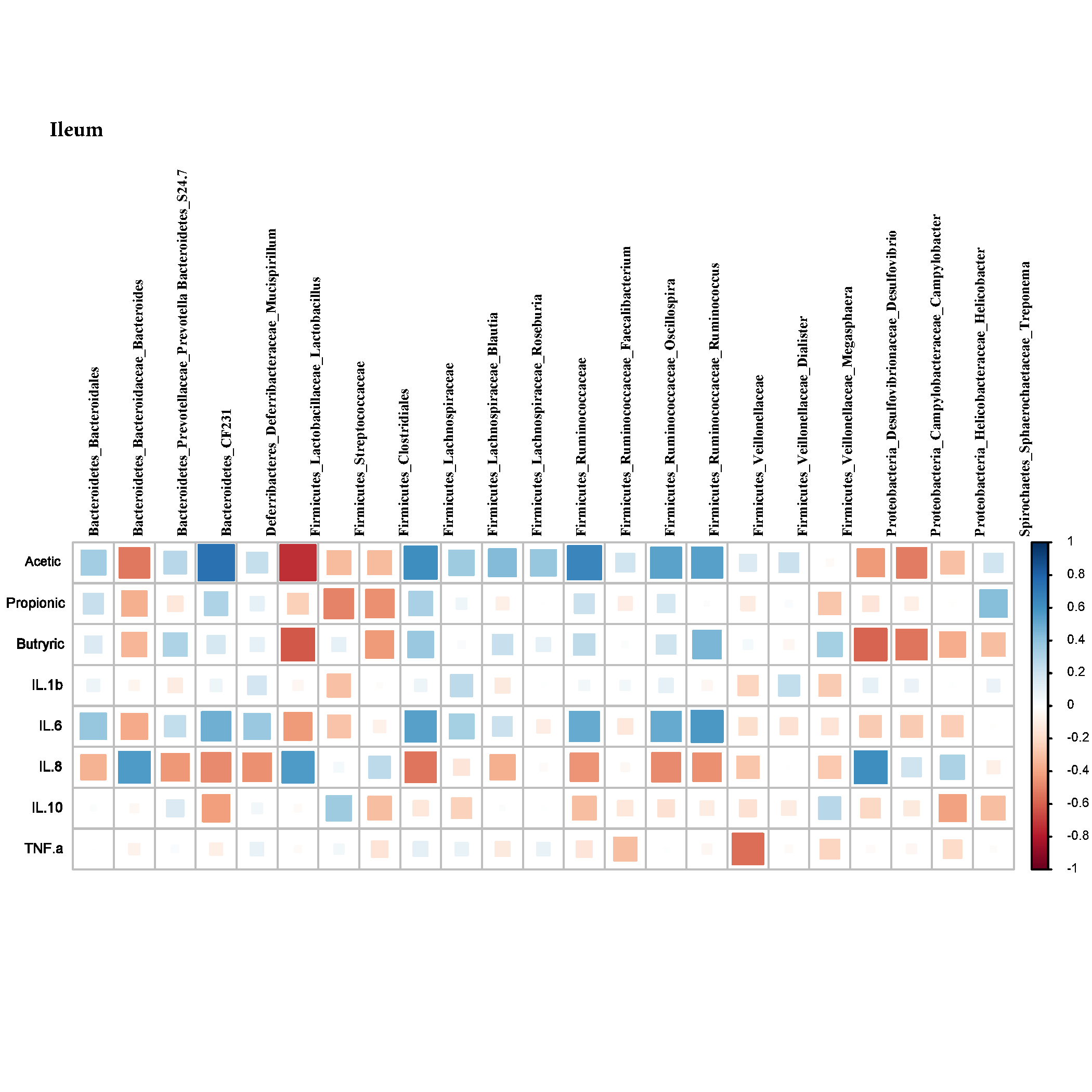

Supplement: Supplementary Figure 1 — Correlation coefficient of selected taxa with the short chain fatty acids (acetic, propionic and butyric acid) and inflammatory markers in the ileum. The blue color shows a positive correlation while the coral color shows a negative correlation. The intensity of the color depicts the strength of the correlation. [file Image1.TIFF]

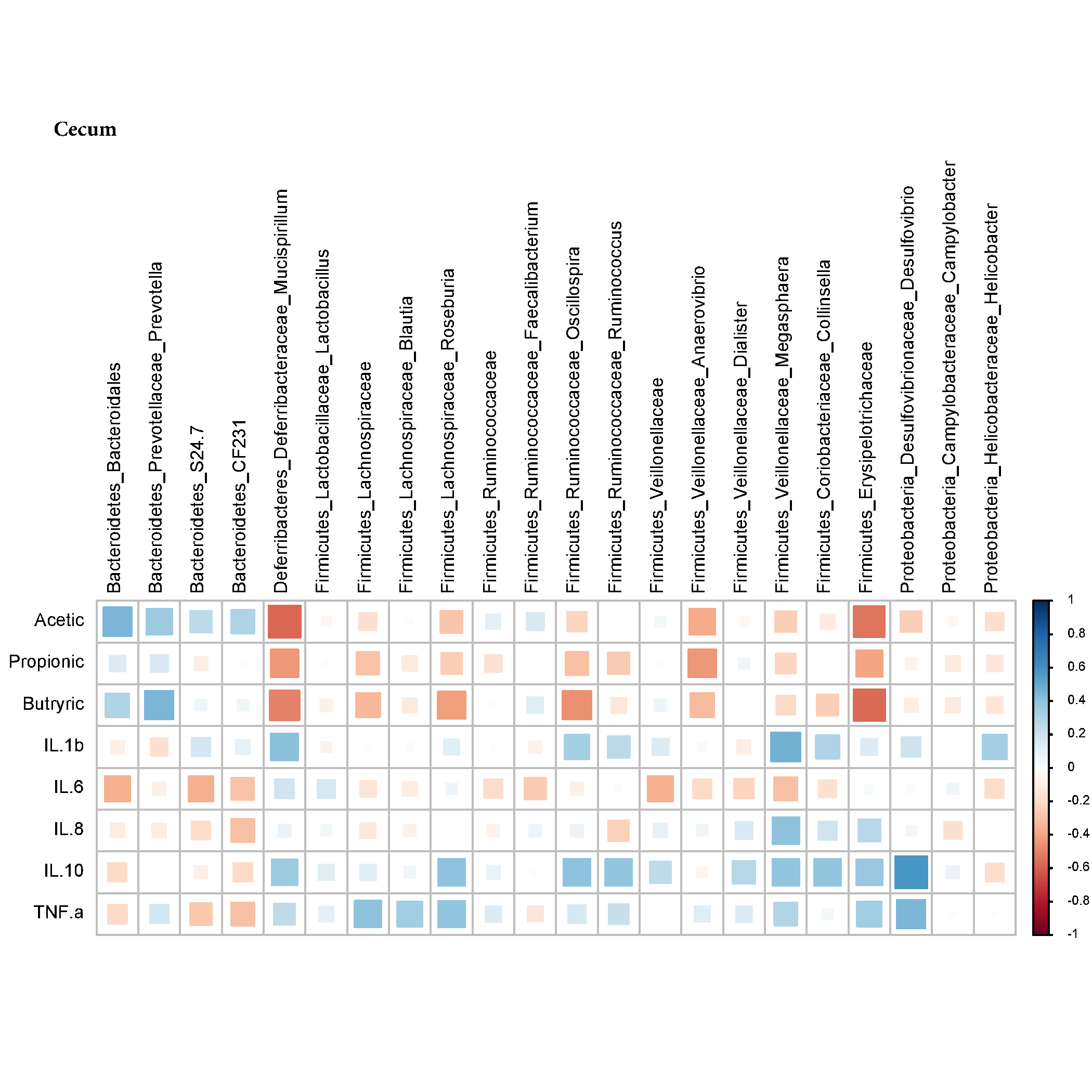

Supplement: Supplementary Figure 2 — Correlation coefficient of selected taxa with the short chain fatty acids (acetic, propionic and butyric acid) and inflammatory markers in the cecum. The blue color shows a positive correlation while the coral color shows a negative correlation. The intensity of the color depicts the strength of the correlation. [file Image2.TIFF]

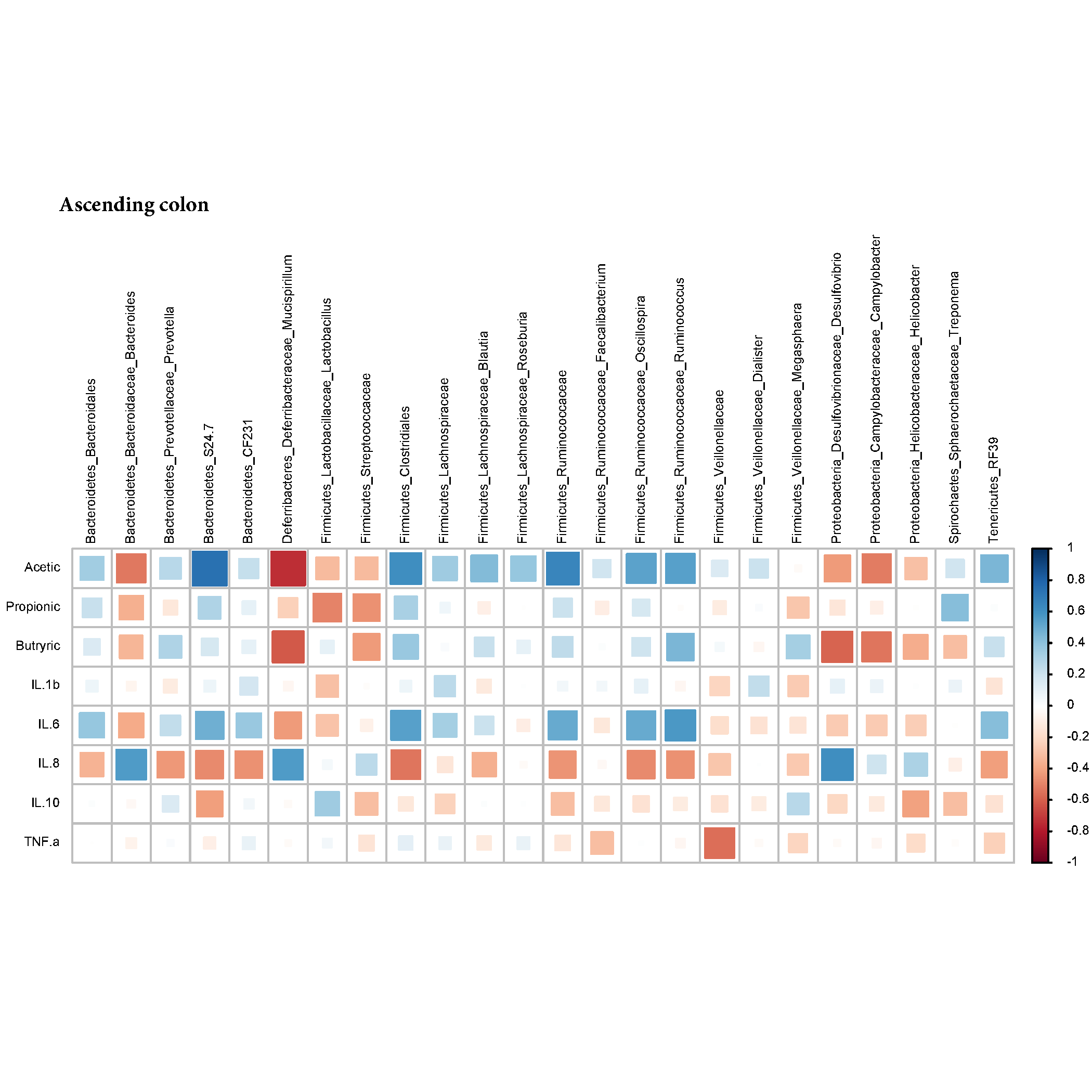

Supplement: Supplementary Figure 3 — Correlation coefficient of selected taxa with the short chain fatty acids (acetic, propionic and butyric acid) and inflammatory markers in the descending colon. The blue color shows a positive correlation while the coral color shows a negative correlation. The intensity of the color depicts the strength of the correlation. [file Image3.TIFF]
