# Supplementary material for: Carrageenan Gum and Adherent Invasive Escherichia coli in a Piglet Model of Inflammatory Bowel Disease: Impact on Intestinal Mucosa-associated Microbiota
Source: Front Microbiol. 2016 Apr 5;7:462. doi: 10.3389/fmicb.2016.00462 (PMC4820460; doi:10.3389/fmicb.2016.00462)
Supplement: Supplementary Table 1 — Analysis of the most abundant phyla in each intestinal segment of pigs treated with carrageenan gum (CG)1 and inoculated with adhered invasive Escherichia coli (AIEC) strain UM1462. [file Table1.DOCX]

| **Supplementary Table 1**. Analysis of the most abundant phyla in each intestinal segment of pigs treated with carrageenan gum; CG^1^ and inoculated with adhered invasive *Escherichia coli* (AIEC) strain UM146^2^. | | | | | | | | |
| --- | --- | --- | --- | --- | --- | --- | --- | --- |
| **Items** | **Treatments** | | | |  |  |  |  |
|  | **UM 146** | **CG** | **CGUM146** | **Control** |  | **SED** |  | ***P value*** |
| **Ileum** |  |  |  |  |  |  |  |  |
| Firmicutes | 76.8695 | 93.3542 | 69.9675 | 73.6225 |  | 12.7 |  | 0.2588 |
| Bacteroidetes | 7.6663 | 2.0287 | 9.4842 | 2.0326 |  | 4.3 |  | 0.1382 |
| Proteobacteria | 8.4468 | 6.4346 | 15.0357 | 10.3429 |  | 2.9 |  | 0.2312 |
| Tenericutes | 0.02587 | 0.0579 | 0.2372 | 0.0899 |  | 0.08 |  | 0.1217 |
| **Cecum** |  |  |  |  |  |  |  |  |
| Firmicutes | 42.0656 | 48.0226 | 36.0631 | 34.9073 |  | 10.8 |  | 0.5668 |
| Bacteroidetes | 35.1657^a^ | 4.7135^c^ | 14.7126^abc^ | 28.961^ab^ |  | 7.8 |  | **0.002** |
| Proteobacteria | 26.2615 | 52.8395 | 51.0098 | 43.4782 |  | 13.5 |  | 0.3568 |
| Deferribacteres | 0.1138 | 0.9278 | 0.7182 | 0.8592 |  | 0.45 |  | 0.3257 |
| **Ascending Colon** |  |  |  |  |  |  |  |  |
| Firmicutes | 77.044^a^ | 48.67^b^ | 40.854^b^ | 55.326^ab^ |  | 8.5 |  | **0.0070** |
| Bacteroidetes | 17.444^ab^ | 5.027^cb^ | 27.3776^a^ | 26.3016^a^ |  | 0.3 |  | **<.0001** |
| Proteobacteria | 3.3882^b^ | 24.2887^a^ | 18.366^a^ | 11.048^b^ |  | 0.2 |  | **0.001** |
| Spirochaetes | 0.4752 | 0.5688 | 1.1368 | 1.1202 |  | 0.6 |  | 0.5348 |
| Deferribacteres | 0.9962^b^ | 2.4868^a^ | 4.246^a^ | 0.3614^b^ |  | 0.3 |  | **0.0393** |
| **Descending Colon** |  |  |  |  |  |  |  |  |
| Firmicutes | 37.4655^ab^ | 30.2227^b^ | 31.0306^b^ | 49.9345^a^ |  | 5.6 |  | **0.0055** |
| Bacteroidetes | 57.268^a^ | 18.967^b^ | 56.162^a^ | 42.38^a^ |  | 5.6 |  | **<.0001** |
| Proteobacteria | 3.2064^b^ | 35.8847^a^ | 7.7043^b^ | 4.2591^b^ |  | 0.4 |  | **<.0001** |
| Tenericutes | 0.688^ab^ | 0.282^b^ | 0.5027^b^ | 0.9955^a^ |  | 0.1 |  | **0.0047** |
| Deferribacteres | 0.05434 | 0.8774 | 1.2915 | 0.03921 |  | 0.5 |  | 0.1201 |
| ^1^Carrageenan gum was administered to the designated groups from d 1 of the experiment. Pigs received 1 % of CG in drinking water on daily basis  ^2^AIEC UM146 inoculation was administered on d 8 of the experiment. Pigs in designated groups received 100 mL of an overnight AIEC UM146 culture (108 CFU/mL) in feed.  CG= pigs received 1% carrageenan gum only in drinking water on daily basis, UM146=pigs were inoculated with AIEC UM146 on d 8 of the study, CGUM146=pigs received CG from d 1 of the study and were infected with AIEC UM146 on d 8 of the study. | | | | | | | | |
