# Supplementary material for: Carrageenan Gum and Adherent Invasive Escherichia coli in a Piglet Model of Inflammatory Bowel Disease: Impact on Intestinal Mucosa-associated Microbiota
Source: Front Microbiol. 2016 Apr 5;7:462. doi: 10.3389/fmicb.2016.00462 (PMC4820460; doi:10.3389/fmicb.2016.00462)
Supplement: Supplementary Table 2 — A summary showing mean relative abundances of taxa in ileal mucosa samples. [file Table2.docx]

| **Supplementary Table 2:** A summary showing mean relative abundances of taxa **in ileal mucosa samples**. While majority of taxa were classified at the genus level (g.), some were only classified at the phylum (p.), class (c.), order (o.), or family (f.) levels. | | | | |
| --- | --- | --- | --- | --- |
| **Taxa** | **Mean relative abundance*** | | | |
|  | **Control** | **UM146** | **CG** | **CGUM146** |
| **--------------------------Greater than or equal to 0.01% of community------------------------** | | | | |
| g. *Corynebacterium* | 0.001 | 0.033 | 0.009 | 0.071 |
| g. *Kocuria* | 0.047 | 0.033 | 0.000 | 0.002 |
| g. *Bifidobacterium* | 0.033 | 0.056 | 0.001 | 0.046 |
| o. Bacteroidales | 0.351 | 1.776 | 0.184 | 1.099 |
| g. *5-7N15* | 0.000 | 0.010 | 0.053 | 0.010 |
| g. *Bacteroides* | 0.009 | 0.586 | 0.042 | 2.388 |
| g. *Parabacteroides* | 0.006 | 0.149 | 0.039 | 0.015 |
| g. *Prevotella* | 1.531 | 4.255 | 1.305 | 5.523 |
| f. Rikenellaceae | 0.000 | 0.008 | 0.013 | 0.036 |
| f. S24-7 | 0.090 | 0.560 | 0.208 | 0.296 |
| g. *CF231* | 0.018 | 0.230 | 0.000 | 0.025 |
| f. Chitinophagaceae | 0.013 | 0.041 | 0.055 | 0.058 |
| g. *Hymenobacter* | 0.000 | 0.000 | 0.109 | 0.000 |
| o. YS2 | 0.015 | 0.008 | 0.013 | 0.015 |
| o. CAB-I | 0.451 | 0.046 | 0.196 | 0.013 |
| o. Streptophyta | 0.001 | 0.005 | 0.003 | 0.095 |
| g. *Mucispirillum* | 0.022 | 0.393 | 0.010 | 0.137 |
| p. Firmicutes | 0.069 | 0.015 | 0.026 | 0.066 |
| f. Staphylococcaceae | 0.000 | 0.043 | 0.290 | 0.003 |
| o. Lactobacillales | 0.014 | 0.037 | 0.009 | 0.006 |
| g. *Enterococcus* | 0.000 | 0.626 | 0.000 | 0.010 |
| f. Enterococcaceae | 0.655 | 0.120 | 0.868 | 1.037 |
| f. Lactobacillaceae | 0.084 | 0.037 | 0.198 | 0.147 |
| g. *Lactobacillus* | 28.560 | 62.010 | 77.631 | 34.800 |
| f. Streptococcaceae | 2.880 | 0.470 | 1.750 | 0.524 |
| g. *Streptococcus* | 0.756 | 4.859 | 0.485 | 0.304 |
| g. *Turicibacter* | 0.029 | 0.015 | 0.033 | 0.019 |
| c. Clostridia | 5.987 | 0.063 | 0.192 | 0.700 |
| o. Clostridiales | 0.092 | 0.151 | 0.162 | 0.462 |
| f. Christensenellaceae | 0.009 | 0.006 | 0.089 | 0.026 |
| f. Clostridiaceae | 0.254 | 0.282 | 0.094 | 16.969 |
| g. *Clostridium* | 0.356 | 0.000 | 0.006 | 0.013 |
| g. *Sarcina* | 12.519 | 0.037 | 1.191 | 0.071 |
| f. Lachnospiraceae | 0.092 | 0.348 | 0.625 | 0.634 |
| g. *Blautia* | 0.206 | 0.512 | 0.567 | 0.492 |
| g. *Butyrivibrio* | 0.015 | 0.012 | 0.007 | 0.007 |
| g. *Coprococcus* | 0.022 | 0.039 | 0.007 | 0.101 |
| g. *Dorea* | 0.076 | 0.165 | 0.337 | 0.137 |
| g. *Lachnospira* | 0.007 | 0.017 | 0.000 | 0.102 |
| g. *Oribacterium* | 0.013 | 0.070 | 0.002 | 0.057 |
| g. *Roseburia* | 0.307 | 0.533 | 0.169 | 0.397 |
| f. Lachnospiraceae | 0.141 | 0.141 | 0.309 | 0.517 |
| f. Peptostreptococcaceae | 9.219 | 0.044 | 0.378 | 0.143 |
| f. Ruminococcaceae | 0.209 | 0.582 | 0.450 | 0.754 |
| f. Ruminococcaceae | 0.297 | 0.408 | 0.629 | 0.484 |
| g. *Faecalibacterium* | 0.174 | 0.278 | 0.216 | 0.482 |
| g. *Oscillospira* | 0.078 | 0.239 | 0.069 | 0.174 |
| g. *Ruminococcus* | 8.490 | 0.445 | 0.685 | 1.432 |
| f. Veillonellaceae | 0.181 | 0.450 | 0.522 | 0.605 |
| g. *Acidaminococcus* | 0.006 | 0.000 | 0.000 | 0.048 |
| g. *Anaerovibrio* | 0.107 | 0.509 | 0.261 | 0.177 |
| g. *Dialister* | 0.693 | 0.448 | 0.734 | 1.877 |
| g. *Megasphaera* | 0.138 | 1.985 | 0.657 | 4.663 |
| g. *Mitsuokella* | 0.057 | 0.158 | 0.006 | 0.506 |
| g. *Phascolarctobacterium* | 0.015 | 0.031 | 0.010 | 0.079 |
| g. *Selenomonas* | 0.003 | 0.155 | 0.000 | 0.048 |
| g. *Veillonella* | 0.493 | 0.274 | 0.023 | 0.347 |
| f. Coriobacteriaceae | 0.024 | 0.023 | 0.100 | 0.024 |
| f. Erysipelotrichaceae | 0.002 | 0.002 | 0.014 | 0.118 |
| g. *Allobaculum* | 0.021 | 0.004 | 0.223 | 0.007 |
| g. *Bulleidia* | 0.058 | 0.033 | 0.019 | 0.106 |
| f. Erysipelotrichaceae | 0.076 | 0.107 | 0.356 | 0.133 |
| o. Erysipelotrichales | 0.001 | 0.000 | 0.039 | 0.000 |
| g. *Catenibacterium* | 0.083 | 0.000 | 0.147 | 0.063 |
| g. *Sharpea* | 0.000 | 0.010 | 0.117 | 0.006 |
| f. Fusobacteriaceae | 0.000 | 0.000 | 0.003 | 0.206 |
| o. RF32 | 0.009 | 0.128 | 0.026 | 0.019 |
| f. Bradyrhizobiaceae | 0.002 | 0.002 | 0.005 | 0.027 |
| g. *Methylobacterium* | 0.000 | 0.000 | 0.002 | 0.035 |
| f. mitochondria | 0.003 | 0.002 | 0.022 | 0.263 |
| g. *Sphingobium* | 0.040 | 0.000 | 0.186 | 0.094 |
| g. *Sphingomonas* | 0.001 | 0.012 | 0.022 | 0.009 |
| c. Betaproteobacteria | 0.017 | 0.077 | 0.334 | 0.019 |
| g. *Sutterella* | 0.052 | 0.263 | 0.086 | 0.793 |
| f. Comamonadaceae | 0.006 | 0.012 | 0.061 | 0.041 |
| g. *Comamonas* | 0.007 | 0.041 | 0.077 | 0.000 |
| g. *Hylemonella* | 0.000 | 0.026 | 0.000 | 0.036 |
| f. Oxalobacteraceae | 0.008 | 0.019 | 0.317 | 0.036 |
| g. *Desulfovibrio* | 0.050 | 0.360 | 0.018 | 0.187 |
| g. *Campylobacte* | 1.691 | 0.482 | 0.366 | 4.433 |
| g. *Helicobacter* | 0.065 | 0.581 | 0.024 | 1.906 |
| f. Succinivibrionaceae | 0.006 | 0.000 | 0.008 | 0.073 |
| g. *Succinivibrio* | 0.049 | 0.128 | 0.141 | 0.112 |
| f. Enterobacteriaceae | 0.014 | 0.391 | 0.571 | 3.634 |
| g. *Enterobacter* | 0.000 | 0.004 | 0.030 | 0.172 |
| g. *Escherichia* | 0.281 | 5.409 | 0.417 | 2.319 |
| f. Pasteurellaceae | 10.343 | 5.803 | 0.904 | 0.199 |
| g. *Actinobacillus* | 0.028 | 0.025 | 0.014 | 0.002 |
| g. *Acinetobacter* | 0.032 | 0.019 | 0.271 | 0.028 |
| f. Pseudomonadaceae | 0.269 | 0.356 | 1.330 | 0.251 |
| g. *Pseudomonas* | 0.004 | 0.004 | 0.026 | 0.017 |
| f. Xanthomonadaceae | 0.051 | 0.050 | 0.780 | 0.217 |
| g. *Treponema* | 0.000 | 0.271 | 0.000 | 0.000 |
| c. Mollicutes | 0.000 | 0.066 | 0.000 | 0.029 |
| g. *RFN20* | 0.008 | 0.023 | 0.000 | 0.015 |
| g. *Mycoplasma* | 10.547 | 0.005 | 0.000 | 4.491 |
| o. RF39 | 0.056 | 0.138 | 0.224 | 0.097 |
| Unclassified | 0.049 | 0.043 | 0.055 | 0.212 |
| **---------------------------------------Less than 0.01% of community------------------------------** | | | | |
| o. Acidimicrobiales | 0.0000 | 0.0000 | 0.0032 | 0.0024 |
| o. Actinomycetales | 0.0017 | 0.0000 | 0.0000 | 0.0094 |
| g. *Microbispora* | 0.0000 | 0.0186 | 0.0000 | 0.0118 |
| g. *Mycobacterium* | 0.0000 | 0.0000 | 0.0000 | 0.0040 |
| f. BS11 | 0.0000 | 0.0020 | 0.0010 | 0.0024 |
| g. *Paludibacter* | 0.0000 | 0.0000 | 0.0000 | 0.0028 |
| g. *Porphyromonas* | 0.0009 | 0.0000 | 0.0032 | 0.0000 |
| g. *Odoribacter* | 0.0000 | 0.0062 | 0.0000 | 0.0033 |
| f. Flavobacteriaceae | 0.0028 | 0.0013 | 0.0000 | 0.0073 |
| f. Pedobacter | 0.0100 | 0.0000 | 0.0134 | 0.0012 |
| c. Bacilli | 0.0028 | 0.0000 | 0.0000 | 0.0008 |
| f. Bacillaceae | 0.0029 | 0.0000 | 0.0063 | 0.0000 |
| g. *Geobacillus* | 0.0028 | 0.0000 | 0.0072 | 0.0019 |
| f. Planococcaceae | 0.0000 | 0.0012 | 0.0010 | 0.0000 |
| g. *Staphylococcus* | 0.0000 | 0.0103 | 0.0065 | 0.0000 |
| g. *Lactococcus* | 0.0028 | 0.0000 | 0.0016 | 0.0000 |
| f. Catabacteriaceae | 0.0166 | 0.0000 | 0.0000 | 0.0017 |
| g. *Peptoniphilus* | 0.0000 | 0.0000 | 0.0058 | 0.0000 |
| g. *Anaerostipes* | 0.0000 | 0.0000 | 0.0099 | 0.0167 |
| g. *Lachnobacterium* | 0.0017 | 0.0000 | 0.0000 | 0.0033 |
| g. *Peptococcus* | 0.0017 | 0.0103 | 0.0167 | 0.0023 |
| g. *rc4-4* | 0.0006 | 0.0116 | 0.0000 | 0.0000 |
| o. Coriobacteriales | 0.0000 | 0.0000 | 0.0000 | 0.0049 |
| g. *Collinsella* | 0.0024 | 0.0021 | 0.0000 | 0.0072 |
| g. p-75-a5 | 0.0055 | 0.0000 | 0.0016 | 0.0000 |
| g. *Coprobacillus* | 0.0048 | 0.0000 | 0.0000 | 0.0165 |
| f. Caulobacteraceae | 0.0078 | 0.0000 | 0.0072 | 0.0123 |
| o. Ellin329 | 0.0018 | 0.0000 | 0.0000 | 0.0049 |
| g. *Bradyrhizobium* | 0.0006 | 0.0000 | 0.0070 | 0.0047 |
| g. *Hyphomicrobium* | 0.0012 | 0.0000 | 0.0164 | 0.0023 |
| f. Rhodospirillaceae | 0.0028 | 0.0000 | 0.0000 | 0.0008 |
| o. Sphingomonadales | 0.0041 | 0.0000 | 0.0000 | 0.0000 |
| g. *Acidovorax* | 0.0116 | 0.0037 | 0.0000 | 0.0000 |
| g. *Janthinobacterium* | 0.0009 | 0.0000 | 0.0130 | 0.0094 |
| g. *Ralstonia* | 0.0009 | 0.0000 | 0.0000 | 0.0094 |
| c. Gammaproteobacteria | 0.0000 | 0.0000 | 0.0000 | 0.0035 |
| g. *Anaerobiospirillum* | 0.0000 | 0.0000 | 0.0000 | 0.0254 |
| g. *Erwinia* | 0.0006 | 0.0128 | 0.0000 | 0.0000 |
| g. *Trabulsiella* | 0.0000 | 0.0041 | 0.0000 | 0.0144 |
| g. Yersinia | 0.0083 | 0.0000 | 0.0065 | 0.0052 |
| g. *Alkanindiges* | 0.0000 | 0.0012 | 0.0261 | 0.0000 |
| f. Sinobacteraceae | 0.0000 | 0.0062 | 0.0020 | 0.0017 |
| g. *Stenotrophomonas* | 0.0000 | 0.0413 | 0.0000 | 0.0035 |
| g. *Candidatus* *cloacamonas* | 0.0000 | 0.0054 | 0.0000 | 0.0071 |
| g. *Akkermansia* | 0.0000 | 0.0041 | 0.0020 | 0.0000 |

* Mean values only, no statistics
