# Supplementary material for: Carrageenan Gum and Adherent Invasive Escherichia coli in a Piglet Model of Inflammatory Bowel Disease: Impact on Intestinal Mucosa-associated Microbiota
Source: Front Microbiol. 2016 Apr 5;7:462. doi: 10.3389/fmicb.2016.00462 (PMC4820460; doi:10.3389/fmicb.2016.00462)
Supplement: Supplementary Table 3 — A summary showing mean relative abundances of taxa in cecal mucosa samples. [file Table3.docx]

| **Supplementary Table 3:** A summary showing mean relative abundances of taxa in **Cecal mucosa samples**. While majority of taxa were classified at the genus level (g.), some were only classified at the phylum (p.), class (c.), order (o.), or family (f.) levels. | | | | |
| --- | --- | --- | --- | --- |
| **Taxa** | **Mean relative abundance*** | | | |
|  | **Control** | **UM146** | **CG** | **CGUM146** |
| **--------------------------------Greater than or equal to 0.01% of community------------------** | | | | |
| g. *Bifidobacterium* | 0.017 | 0.039 | 0.303 | 0.007 |
| o. Bacteroidales | 0.344 | 0.347 | 0.026 | 0.088 |
| o. Bacteroidales | 1.546 | 0.952 | 0.261 | 0.608 |
| g. *Bacteroides* | 0.014 | 0.017 | 0.424 | 4.329 |
| g. *Parabacteroides* | 0.147 | 0.084 | 0.038 | 0.050 |
| g. *Prevotella* | 12.610 | 16.819 | 1.138 | 7.683 |
| f. S24-7 | 3.570 | 0.587 | 0.185 | 0.308 |
| g. *Butyricimonas* | 0.154 | 0.000 | 0.000 | 0.000 |
| g. *CF231* | 1.238 | 0.541 | 0.240 | 0.124 |
| o. Bacteroidales | 9.293 | 10.022 | 2.180 | 2.964 |
| o. YS2 | 0.031 | 0.009 | 0.015 | 0.014 |
| g. *Mucispirillum* | 2.067 | 0.097 | 1.836 | 2.739 |
| p. Firmicutes | 0.136 | 0.154 | 0.149 | 0.113 |
| o. Lactobacillales | 0.008 | 0.006 | 0.058 | 0.007 |
| f. Lactobacillaceae | 0.080 | 0.089 | 0.469 | 0.327 |
| f. Lactobacillaceae | 0.013 | 0.025 | 0.110 | 0.062 |
| g. *Lactobacillus* | 5.845 | 24.427 | 24.886 | 8.046 |
| f. Streptococcaceae | 0.044 | 0.065 | 0.734 | 0.250 |
| f. Streptococcaceae | 0.000 | 0.000 | 0.064 | 0.004 |
| g. *Streptococcus* | 0.028 | 0.058 | 0.209 | 0.143 |
| g. *Turicibacter* | 0.020 | 0.025 | 0.036 | 0.006 |
| c. Clostridia | 0.175 | 0.162 | 0.076 | 0.105 |
| c. Clostridia | 0.041 | 0.105 | 0.051 | 0.172 |
| o. Clostridiales | 0.112 | 0.127 | 0.075 | 0.064 |
| f. Christensenellaceae | 0.007 | 0.021 | 0.010 | 0.007 |
| f. Clostridiaceae | 0.056 | 0.058 | 0.131 | 0.061 |
| f. Clostridiaceae | 0.096 | 0.116 | 0.138 | 0.223 |
| g. *Clostridium* | 0.029 | 0.002 | 0.006 | 0.006 |
| g. *Sarcina* | 0.957 | 0.032 | 0.158 | 0.088 |
| f. Lachnospiraceae | 0.545 | 0.757 | 0.550 | 0.560 |
| f. Lachnospiraceae | 0.046 | 0.110 | 0.067 | 0.089 |
| g. *Blautia* | 0.812 | 1.083 | 0.818 | 0.525 |
| g. *Butyrivibrio* | 0.007 | 0.015 | 0.026 | 0.012 |
| g. *Coprococcus* | 0.169 | 0.116 | 0.144 | 0.228 |
| g. *Dorea* | 0.204 | 0.378 | 0.274 | 0.171 |
| g. *Lachnospira* | 0.020 | 0.062 | 0.024 | 0.030 |
| g. *Oribacterium* | 0.089 | 0.074 | 0.023 | 0.069 |
| g. *Roseburia* | 1.250 | 1.644 | 1.984 | 1.559 |
| f. Lachnospiraceae | 0.608 | 0.620 | 0.561 | 0.529 |
| g. *Peptococcus* | 0.026 | 0.046 | 0.033 | 0.030 |
| f. Peptostreptococcaceae | 0.142 | 0.206 | 0.333 | 0.069 |
| f. Ruminococcaceae | 4.286 | 4.060 | 3.313 | 3.728 |
| g. *Anaerotruncus* | 0.019 | 0.002 | 0.010 | 0.009 |
| g. *Faecalibacterium* | 3.227 | 2.887 | 1.155 | 0.786 |
| g. *Oscillospira* | 1.077 | 0.410 | 0.768 | 0.540 |
| g. *Ruminococcus* | 1.213 | 0.934 | 0.649 | 1.523 |
| f. Veillonellaceae | 1.891 | 3.925 | 1.183 | 1.217 |
| g. *Acidaminococcus* | 0.689 | 0.408 | 0.174 | 0.129 |
| g. *Anaerovibrio* | 0.541 | 0.317 | 0.507 | 0.639 |
| g. *Dialister* | 3.106 | 3.954 | 1.508 | 3.382 |
| g. *Megasphaera* | 0.996 | 2.593 | 1.652 | 4.136 |
| g. *Mitsuokella* | 0.357 | 0.219 | 0.162 | 0.152 |
| g. *Phascolarctobacterium* | 0.181 | 0.318 | 0.057 | 0.153 |
| g. *Selenomonas* | 0.005 | 0.108 | 0.023 | 0.013 |
| g. *Veillonella* | 0.005 | 0.005 | 0.005 | 0.026 |
| f. Coriobacteriaceae | 0.721 | 0.013 | 0.466 | 0.025 |
| g. *Collinsella* | 0.029 | 0.010 | 1.484 | 0.018 |
| g. *Slackia* | 0.013 | 0.000 | 0.110 | 0.000 |
| g. *Bulleidia* | 0.100 | 0.170 | 0.101 | 0.075 |
| f. Erysipelotrichaceae | 0.361 | 0.282 | 0.934 | 0.554 |
| g. *p-75-a5* | 0.041 | 0.012 | 0.029 | 0.011 |
| g. *Catenibacterium* | 0.334 | 0.038 | 0.213 | 0.275 |
| f. Fusobacteriaceae | 0.004 | 0.000 | 0.000 | 0.107 |
| f. Mitochondria | 0.000 | 0.001 | 0.013 | 0.056 |
| g. *Sutterella* | 0.114 | 0.115 | 0.047 | 0.556 |
| g. *Hylemonella* | 0.000 | 0.000 | 0.085 | 0.000 |
| f. Desulfovibrionaceae | 0.006 | 0.017 | 0.027 | 0.009 |
| g. *Desulfovibrio* | 0.896 | 0.819 | 0.685 | 0.721 |
| o. Campylobacterales | 0.070 | 0.063 | 0.230 | 0.146 |
| g. *Campylobacter* | 4.640 | 12.429 | 7.898 | 23.385 |
| f. Helicobacteraceae | 0.013 | 0.004 | 0.005 | 0.029 |
| g. *Helicobacter* | 31.140 | 4.443 | 36.639 | 23.696 |
| f. Succinivibrionaceae | 0.002 | 0.002 | 0.025 | 0.035 |
| g. *Anaerobiospirillum* | 0.082 | 0.034 | 0.041 | 0.017 |
| g. *Succinivibrio* | 0.029 | 0.279 | 0.068 | 0.070 |
| f. Enterobacteriaceae | 0.012 | 0.005 | 0.030 | 0.328 |
| g. *Escherichia* | 0.101 | 0.049 | 0.043 | 0.137 |
| f. Pasteurellaceae | 0.140 | 0.018 | 0.011 | 0.001 |
| f. Pseudomonadaceae | 0.008 | 0.049 | 0.036 | 0.016 |
| f. Xanthomonadaceae | 0.006 | 0.015 | 0.018 | 0.002 |
| g. *Treponema* | 0.151 | 0.186 | 0.015 | 0.045 |
| g. *RFN20* | 0.059 | 0.047 | 0.139 | 0.022 |
| g. *Mycoplasma* | 0.007 | 0.000 | 0.000 | 0.147 |
| o. RF39 | 0.244 | 0.236 | 0.156 | 0.230 |
| Unclassified | 0.271 | 0.267 | 0.165 | 0.225 |
| **-----------------------------------------------Less than 0.01% of community----------------------** | | | | |
| g. *Kocuria* | 0.0018 | 0.0038 | 0.0075 | 0.0000 |
| f. Nocardioidaceae | 0.0011 | 0.0000 | 0.0025 | 0.0008 |
| p. Bacteroidetes | 0.0128 | 0.0044 | 0.0014 | 0.0078 |
| f. BS11 | 0.0025 | 0.0000 | 0.0026 | 0.0007 |
| f. Porphyromonadaceae | 0.0117 | 0.0013 | 0.0000 | 0.0000 |
| g. *Paludibacter* | 0.0018 | 0.0016 | 0.0010 | 0.0000 |
| f. Prevotellaceae | 0.0026 | 0.0000 | 0.0000 | 0.0000 |
| f. Rikenellaceae | 0.0068 | 0.0000 | 0.0007 | 0.0007 |
| g. YRC22 | 0.0032 | 0.0020 | 0.0015 | 0.0029 |
| f. Flavobacteriaceae | 0.0004 | 0.0000 | 0.0088 | 0.0004 |
| f. Chitinophagaceae | 0.0010 | 0.0000 | 0.0012 | 0.0000 |
| g. *Pedobacter* | 0.0032 | 0.0000 | 0.0049 | 0.0004 |
| f. Chlamydiaceae | 0.0000 | 0.0000 | 0.0020 | 0.0021 |
| o. CAB-I | 0.0000 | 0.0023 | 0.0031 | 0.0012 |
| o. Streptophyta | 0.0000 | 0.0000 | 0.0105 | 0.0028 |
| f. Elusimicrobiaceae | 0.0009 | 0.0024 | 0.0000 | 0.0000 |
| c. Bacilli | 0.0000 | 0.0000 | 0.0003 | 0.0033 |
| f. Planococcaceae | 0.0000 | 0.0023 | 0.0021 | 0.0000 |
| g. *Lactococcus* | 0.0021 | 0.0023 | 0.0000 | 0.0000 |
| f. Catabacteriaceae | 0.0059 | 0.0000 | 0.0117 | 0.0004 |
| g. *Anaerostipes* | 0.0038 | 0.0132 | 0.0052 | 0.0021 |
| g. *Lachnobacterium* | 0.0018 | 0.0000 | 0.0100 | 0.0008 |
| o. Clostridiales | 0.0037 | 0.0000 | 0.0000 | 0.0000 |
| g. *Olsenella* | 0.0063 | 0.0000 | 0.0015 | 0.0024 |
| g. *Allobaculum* | 0.0177 | 0.0015 | 0.0136 | 0.0008 |
| g. *L7A_E11* | 0.0021 | 0.0000 | 0.0003 | 0.0000 |
| g. *cc_115* | 0.0021 | 0.0000 | 0.0013 | 0.0000 |
| g. *Coprobacillus* | 0.0039 | 0.0000 | 0.0096 | 0.0000 |
| g. *Sharpea* | 0.0115 | 0.0145 | 0.0096 | 0.0000 |
| f. Victivallaceae | 0.0021 | 0.0006 | 0.0000 | 0.0000 |
| c. OP8_1 | 0.0008 | 0.0000 | 0.0015 | 0.0000 |
| p. Proteobacteria | 0.0144 | 0.0040 | 0.0013 | 0.0108 |
| o. RF32 | 0.0148 | 0.0000 | 0.0030 | 0.0008 |
| g. *Methyloba* | 0.0000 | 0.0000 | 0.0005 | 0.0008 |
| g. Paracoc | 0.0042 | 0.0000 | 0.0046 | 0.0000 |
| f. Sphingomonadaceae | 0.0000 | 0.0000 | 0.0050 | 0.0017 |
| g. *Sphingobium* | 0.0000 | 0.0008 | 0.0200 | 0.0008 |
| g. *Sphingomonas* | 0.0000 | 0.0000 | 0.0074 | 0.0008 |
| c. Betaproteobacteria | 0.0014 | 0.0029 | 0.0005 | 0.0000 |
| c. Betaproteobacteria | 0.0034 | 0.0000 | 0.0012 | 0.0000 |
| o. Burkholderiales | 0.0004 | 0.0008 | 0.0000 | 0.0059 |
| f. Comamonadaceae | 0.0011 | 0.0000 | 0.0050 | 0.0000 |
| g. *Acidovorax* | 0.0011 | 0.0000 | 0.0060 | 0.0008 |
| f. Oxalobacteraceae | 0.0034 | 0.0000 | 0.0062 | 0.0000 |
| g. *Oxalobacter* | 0.0023 | 0.0045 | 0.0024 | 0.0000 |
| g. *Ralstonia* | 0.0000 | 0.0000 | 0.0000 | 0.0000 |
| c. Deltaproteobacteria | 0.0000 | 0.0016 | 0.0103 | 0.0000 |
| o. Desulfovibrionales | 0.0011 | 0.0000 | 0.0000 | 0.0022 |
| o. GMD14H09 | 0.0204 | 0.0098 | 0.0017 | 0.0017 |
| f. Syntrophaceae | 0.0000 | 0.0000 | 0.0012 | 0.0000 |
| f. Campylobacteraceae | 0.0000 | 0.0000 | 0.0007 | 0.0018 |
| c. Gammaproteobacteria | 0.0000 | 0.0000 | 0.0000 | 0.0015 |
| g. *Enterobacter* | 0.0000 | 0.0000 | 0.0006 | 0.0229 |
| g. *Yersinia* | 0.0000 | 0.0023 | 0.0000 | 0.0004 |
| g. *Actinobacillus* | 0.0046 | 0.0016 | 0.0000 | 0.0014 |
| g. *Acinetobacter* | 0.0000 | 0.0093 | 0.0095 | 0.0000 |
| g. *Enhydrobacter* | 0.0000 | 0.0006 | 0.0010 | 0.0004 |
| g. *Brachyspira* | 0.0013 | 0.0000 | 0.0007 | 0.0019 |
| f. Dethiosulfovibrionaceae | 0.0009 | 0.0058 | 0.0000 | 0.0000 |
| c. Mollicutes | 0.0052 | 0.0258 | 0.0000 | 0.0036 |
| f. Anaeroplasmataceae | 0.0018 | 0.0000 | 0.0000 | 0.0042 |
| * Mean values only, no statistics | | | | |
