# Supplementary material for: Carrageenan Gum and Adherent Invasive Escherichia coli in a Piglet Model of Inflammatory Bowel Disease: Impact on Intestinal Mucosa-associated Microbiota
Source: Front Microbiol. 2016 Apr 5;7:462. doi: 10.3389/fmicb.2016.00462 (PMC4820460; doi:10.3389/fmicb.2016.00462)
Supplement: Supplementary Table 4 — A summary showing mean relative abundances of taxa in the ascending colon mucosa samples. [file Table4.docx]

| **Supplementary Table 4:** A summary showing mean relative abundances of taxa in the **Ascending colon** **mucosa samples**. While majority of taxa were classified at the genus level (g.), some were only classified at the phylum (p.), class (c.), order (o.), or family (f.) levels. | | | | |
| --- | --- | --- | --- | --- |
| **Taxa** | **Mean relative abundance*** | | | |
|  | **Control** | **UM146** | **CG** | **CGUM146** |
| **---------------------------Greater than or equal to 0.01% of community----------------** | | | | |
| g. *Bifidobacterium* | 0.121 | 1.074 | 0.032 | 0.100 |
| p. Bacteroidetes | 0.030 | 0.026 | 0.014 | 0.006 |
| o. Bacteroidales | 2.752 | 1.649 | 0.634 | 1.573 |
| g. *Bacteroides* | 0.124 | 0.007 | 0.345 | 8.367 |
| g. *Parabacteroides* | 0.176 | 0.057 | 0.115 | 0.419 |
| g. *Prevotella* | 8.900 | 4.936 | 1.770 | 6.340 |
| f. S24-7 | 1.542 | 3.125 | 1.980 | 2.219 |
| g. *Butyricimonas* | 0.039 | 0.037 | 0.013 | 0.005 |
| g. CF231 | 1.557 | 0.370 | 0.115 | 0.256 |
| o. Bacteroidales | 7.419 | 7.211 | 3.549 | 5.127 |
| o. YS2 | 0.025 | 0.018 | 0.001 | 0.003 |
| o. Streptophyta | 0.003 | 0.004 | 0.018 | 0.017 |
| g. *Mucispirillum* | 6.103 | 0.996 | 17.015 | 10.147 |
| p. Firmicutes | 0.209 | 0.312 | 0.150 | 0.079 |
| f. Lactobacillaceae | 0.221 | 0.184 | 0.409 | 0.432 |
| g. *Lactobacillus* | 11.393 | 24.692 | 13.832 | 9.541 |
| f. Streptococcaceae | 0.181 | 0.031 | 0.560 | 0.165 |
| g. *Streptococcus* | 0.237 | 0.110 | 0.382 | 0.229 |
| c. Clostridia | 0.353 | 0.298 | 0.241 | 0.564 |
| o. Clostridiales | 0.317 | 0.249 | 0.260 | 0.153 |
| f. Catabacteriaceae | 0.045 | 0.052 | 0.057 | 0.001 |
| f. Christensenellaceae | 0.015 | 0.031 | 0.029 | 0.012 |
| f. Clostridiaceae | 0.503 | 0.446 | 0.275 | 0.490 |
| g. *Sarcina* | 2.269 | 0.004 | 0.109 | 0.082 |
| f. Lachnospiraceae | 0.965 | 0.676 | 0.668 | 0.640 |
| g. *Blautia* | 1.454 | 2.027 | 0.841 | 0.438 |
| g. *Coprococcus* | 0.251 | 0.411 | 0.160 | 0.180 |
| g. *Dorea* | 0.351 | 0.480 | 0.327 | 0.070 |
| g. *Lachnospira* | 0.030 | 0.043 | 0.003 | 0.015 |
| g. *Oribacterium* | 0.119 | 0.057 | 0.012 | 0.063 |
| g. *Roseburia* | 4.122 | 1.295 | 11.812 | 1.146 |
| f. Lachnospiraceae | 0.788 | 1.060 | 0.646 | 0.333 |
| g. *Peptococcus* | 0.071 | 0.124 | 0.098 | 0.023 |
| f. Peptostreptococcaceae | 0.047 | 0.031 | 0.019 | 0.003 |
| f. Ruminococcaceae | 6.169 | 12.296 | 4.988 | 6.083 |
| g. *Anaerotruncus* | 0.073 | 0.038 | 0.034 | 0.032 |
| g. *Faecalibacterium* | 6.025 | 7.007 | 2.036 | 3.589 |
| g. *Oscillospira* | 1.785 | 2.768 | 2.177 | 3.127 |
| g. *Ruminococcus* | 2.187 | 1.892 | 3.029 | 2.850 |
| f. Veillonellaceae | 5.833 | 5.066 | 0.603 | 1.371 |
| g. *Acidaminococcus* | 0.375 | 0.375 | 0.138 | 0.129 |
| g. *Anaerovibrio* | 0.575 | 0.601 | 0.552 | 0.158 |
| g. *Dialister* | 4.845 | 5.050 | 0.619 | 1.338 |
| g. *Megasphaera* | 1.046 | 1.947 | 1.014 | 7.425 |
| g. *Mitsuokella* | 0.461 | 0.434 | 0.134 | 0.221 |
| g. *Phascolarctobacterium* | 0.487 | 0.413 | 0.125 | 0.169 |
| g. *Selenomonas* | 0.012 | 0.015 | 0.014 | 0.015 |
| g. *Veillonella* | 0.020 | 0.003 | 0.006 | 0.028 |
| f. Coriobacteriaceae | 0.103 | 0.718 | 0.379 | 0.201 |
| g. *Collinsella* | 0.055 | 0.451 | 0.872 | 0.185 |
| g. *Slackia* | 0.003 | 0.011 | 0.054 | 0.005 |
| g. *Bulleidia* | 0.237 | 0.416 | 0.189 | 0.121 |
| f. Erysipelotrichaceae | 0.411 | 0.563 | 0.469 | 0.447 |
| g. *p-75-a5* | 0.033 | 0.502 | 0.194 | 0.171 |
| g. *Catenibacterium* | 0.578 | 0.145 | 0.101 | 0.166 |
| f. Fusobacteriaceae | 0.000 | 0.000 | 0.013 | 0.034 |
| c. Betaproteobacteria | 0.012 | 0.005 | 0.005 | 0.038 |
| g. *Sutterella* | 0.112 | 0.108 | 0.103 | 0.953 |
| f. Oxalobacteraceae | 0.008 | 0.014 | 0.017 | 0.025 |
| g. *Ralstonia* | 0.012 | 0.024 | 0.029 | 0.084 |
| f. Desulfovibrionaceae | 0.032 | 0.019 | 0.021 | 0.048 |
| g. *Desulfovibrio* | 3.649 | 1.266 | 1.659 | 6.110 |
| o. GMD14H09 | 0.032 | 0.016 | 0.001 | 0.003 |
| o. Campylobacterales | 0.020 | 0.000 | 0.042 | 0.015 |
| g. *Campylobacter* | 0.846 | 1.055 | 3.720 | 4.775 |
| g. *Helicobacter* | 5.272 | 0.553 | 18.068 | 2.696 |
| f. Succinivibrionaceae | 0.009 | 0.002 | 0.067 | 0.047 |
| g. *Anaerobiospirillum* | 0.030 | 0.002 | 0.108 | 0.003 |
| g. *Succinivibrio* | 0.117 | 0.033 | 0.022 | 0.046 |
| f. Enterobacteriaceae | 0.029 | 0.008 | 0.065 | 0.417 |
| g. *Escherichia* | 0.447 | 0.111 | 0.009 | 0.232 |
| f. Pasteurellaceae | 0.220 | 0.003 | 0.004 | 0.004 |
| g. *Acinetobacter* | 0.005 | 0.009 | 0.023 | 0.148 |
| f. Pseudomonadaceae | 0.076 | 0.093 | 0.166 | 0.566 |
| g. *Pseudomonas* | 0.004 | 0.002 | 0.004 | 0.079 |
| f. Xanthomonadaceae | 0.029 | 0.023 | 0.044 | 0.151 |
| g. *Treponema* | 3.892 | 2.194 | 0.554 | 3.307 |
| g. *Brachyspira* | 0.051 | 0.002 | 0.003 | 0.054 |
| g. *RFN20* | 0.041 | 0.038 | 0.048 | 0.073 |
| g. *Mycoplasma* | 0.014 | 0.000 | 0.000 | 0.077 |
| o. RF39 | 0.261 | 0.914 | 0.433 | 1.490 |
| Unclassified | 0.468 | 0.334 | 0.250 | 0.973 |
| **---------------------------------------Less than 0.01% of community------------------------** | | | | |
| o. Acidimicrobiales | 0.0033 | 0.0007 | 0.0010 | 0.0147 |
| o. Actinomycetales | 0.0000 | 0.0025 | 0.0000 | 0.0016 |
| g. *Parascardovia* | 0.0000 | 0.0000 | 0.0010 | 0.0007 |
| g. *Corynebacterium* | 0.0000 | 0.0000 | 0.0057 | 0.0007 |
| f. Geodermatophilaceae | 0.0015 | 0.0000 | 0.0044 | 0.0272 |
| f. Micrococcaceae | 0.0000 | 0.0000 | 0.0053 | 0.0000 |
| g. *Kocuria* | 0.0003 | 0.0005 | 0.0044 | 0.0000 |
| f. Nocardioidaceae | 0.0000 | 0.0000 | 0.0027 | 0.0125 |
| f. BS11 | 0.0003 | 0.0062 | 0.0097 | 0.0002 |
| f. Bacteroidaceae | 0.0005 | 0.0000 | 0.0000 | 0.0062 |
| g. *5-7N15* | 0.0000 | 0.0000 | 0.0015 | 0.0000 |
| f. Marinilabiaceae | 0.0000 | 0.0015 | 0.0057 | 0.0000 |
| f. Porphyromonadaceae | 0.0358 | 0.0004 | 0.0005 | 0.0000 |
| g. *Paludibacter* | 0.0036 | 0.0000 | 0.0057 | 0.0000 |
| f. Prevotellaceae | 0.0019 | 0.0009 | 0.0004 | 0.0000 |
| f. Rikenellaceae | 0.0019 | 0.0064 | 0.0075 | 0.0052 |
| g. *YRC22* | 0.0146 | 0.0026 | 0.0099 | 0.0011 |
| f. p-2534-18B5 | 0.0009 | 0.0010 | 0.0013 | 0.0000 |
| f. Flavobacteriaceae | 0.0005 | 0.0012 | 0.0000 | 0.0070 |
| f. Chitinophagaceae | 0.0000 | 0.0035 | 0.0018 | 0.0076 |
| g. *Hymenobacter* | 0.0000 | 0.0000 | 0.0032 | 0.0000 |
| f. Sphingobacteriaceae | 0.0000 | 0.0000 | 0.0004 | 0.0010 |
| g. *Pedobacter* | 0.0000 | 0.0017 | 0.0087 | 0.0076 |
| o. Chlamydiales | 0.0000 | 0.0000 | 0.0030 | 0.0057 |
| o. CAB-I | 0.0014 | 0.0050 | 0.0075 | 0.0000 |
| c. Bacilli | 0.0026 | 0.0012 | 0.0016 | 0.0068 |
| g. *Staphylococcus* | 0.0014 | 0.0000 | 0.0017 | 0.0020 |
| o. Lactobacillales | 0.0087 | 0.0101 | 0.0103 | 0.0000 |
| g. *Enterococcus* | 0.0008 | 0.0017 | 0.0000 | 0.0014 |
| g. *Leuconostoc* | 0.0000 | 0.0000 | 0.0029 | 0.0010 |
| g. *Lactococcus* | 0.0005 | 0.0017 | 0.0032 | 0.0007 |
| g. *Turicibacter* | 0.0003 | 0.0000 | 0.0003 | 0.0000 |
| g. *Clostridium* | 0.0042 | 0.0059 | 0.0006 | 0.0077 |
| g. *Dehalobacterium* | 0.0010 | 0.0038 | 0.0045 | 0.0000 |
| g. *Anaerostipes* | 0.0026 | 0.0291 | 0.0000 | 0.0000 |
| g. *Butyrivibrio* | 0.0093 | 0.0007 | 0.0018 | 0.0021 |
| g. *Lachnobacterium* | 0.0114 | 0.0000 | 0.0016 | 0.0000 |
| o. Coriobacteriales | 0.0054 | 0.0007 | 0.0039 | 0.0000 |
| g. *Olsenella* | 0.0000 | 0.0017 | 0.0015 | 0.0000 |
| o. Erysipelotrichales | 0.0044 | 0.0021 | 0.0064 | 0.0000 |
| g. *Erysipelothrix* | 0.0018 | 0.0004 | 0.0000 | 0.0009 |
| g. *Holdemania* | 0.0003 | 0.0000 | 0.0046 | 0.0031 |
| g. *L7A_E11* | 0.0029 | 0.0179 | 0.0039 | 0.0144 |
| o. Erysipelotrichales | 0.0011 | 0.0000 | 0.0005 | 0.0013 |
| g. *Sharpea* | 0.0049 | 0.0262 | 0.0054 | 0.0009 |
| o. ML615J-28 | 0.0013 | 0.0056 | 0.0010 | 0.0000 |
| c. Gemm-3 | 0.0000 | 0.0000 | 0.0000 | 0.0040 |
| f. Victivallaceae | 0.0008 | 0.0049 | 0.0000 | 0.0000 |
| f. Pirellulaceae | 0.0008 | 0.0035 | 0.0002 | 0.0000 |
| p. Proteobacteria | 0.0090 | 0.0024 | 0.0129 | 0.0112 |
| f. Caulobacteraceae | 0.0000 | 0.0000 | 0.0008 | 0.0005 |
| o. Ellin329 | 0.0026 | 0.0000 | 0.0034 | 0.0144 |
| o. RF32 | 0.0132 | 0.0087 | 0.0000 | 0.0025 |
| f. Bradyrhizobiaceae | 0.0071 | 0.0000 | 0.0046 | 0.0149 |
| f. Methylobacteriaceae | 0.0000 | 0.0000 | 0.0000 | 0.0048 |
| o. Sphingomonadales | 0.0005 | 0.0000 | 0.0011 | 0.0024 |
| f. Erythrobacteraceae | 0.0000 | 0.0020 | 0.0000 | 0.0000 |
| f. Sphingomonadaceae | 0.0014 | 0.0000 | 0.0060 | 0.0009 |
| g. *Kaistobacter* | 0.0000 | 0.0000 | 0.0008 | 0.0020 |
| g. *Novosphingobium* | 0.0008 | 0.0000 | 0.0006 | 0.0000 |
| g. *Sphingobium* | 0.0000 | 0.0012 | 0.0089 | 0.0222 |
| G. Sphingomonas | 0.0000 | 0.0023 | 0.0050 | 0.0082 |
| o. Burkholderiales | 0.0055 | 0.0007 | 0.0008 | 0.0103 |
| f. Alcaligenaceae | 0.0006 | 0.0000 | 0.0011 | 0.0003 |
| f. Comamonadaceae | 0.0027 | 0.0000 | 0.0028 | 0.0244 |
| g. *Acidovorax* | 0.0000 | 0.0000 | 0.0010 | 0.0164 |
| g. *Comamonas* | 0.0021 | 0.0000 | 0.0033 | 0.0166 |
| g. *Janthinobacterium* | 0.0023 | 0.0000 | 0.0080 | 0.0000 |
| g. *Oxalobacter* | 0.0188 | 0.0057 | 0.0070 | 0.0008 |
| c. Deltaproteobacteria | 0.0040 | 0.0048 | 0.0024 | 0.0005 |
| f. Bacteriovoracaceae | 0.0010 | 0.0000 | 0.0011 | 0.0005 |
| o. Desulfovibrionales | 0.0077 | 0.0009 | 0.0008 | 0.0084 |
| g. *Bilophila* | 0.0000 | 0.0014 | 0.0000 | 0.0000 |
| o. Myxococcales | 0.0000 | 0.0000 | 0.0008 | 0.0005 |
| f. Campylobacteraceae | 0.0000 | 0.0000 | 0.0012 | 0.0002 |
| f. Helicobacteraceae | 0.0023 | 0.0000 | 0.0066 | 0.0009 |
| c. Gammaproteobacteria | 0.0018 | 0.0022 | 0.0032 | 0.0102 |
| g. *Enterobacter* | 0.0000 | 0.0000 | 0.0000 | 0.0009 |
| g. *Erwinia* | 0.0000 | 0.0000 | 0.0000 | 0.0060 |
| g. *Yersinia* | 0.0003 | 0.0000 | 0.0000 | 0.0108 |
| g. *Alkanindiges* | 0.0000 | 0.0037 | 0.0006 | 0.0060 |
| f. Sinobacteraceae | 0.0000 | 0.0000 | 0.0014 | 0.0040 |
| g. *Lysobacter* | 0.0005 | 0.0000 | 0.0000 | 0.0151 |
| c. Spirochaetes | 0.0017 | 0.0000 | 0.0004 | 0.0002 |
| g. *Sphaerochaeta* | 0.0003 | 0.0000 | 0.0013 | 0.0004 |
| f. Sphaerochaetaceae | 0.0013 | 0.0007 | 0.0002 | 0.0012 |
| g. *Candidatus Cloacamonas* | 0.0000 | 0.0000 | 0.0095 | 0.0235 |
| f. Dethiosulfovibrionaceae | 0.0081 | 0.0076 | 0.0000 | 0.0000 |
| f. F16 | 0.0000 | 0.0008 | 0.0032 | 0.0000 |
| c. Mollicutes | 0.0038 | 0.0199 | 0.0072 | 0.0073 |
| f. Anaeroplasmataceae | 0.0009 | 0.0000 | 0.0004 | 0.0012 |
| g. *Deinococcus* | 0.0015 | 0.0000 | 0.0000 | 0.0014 |
| f. RFP12 | 0.0005 | 0.0004 | 0.0004 | 0.0000 |
| g. *Akkermansia* | 0.0000 | 0.0009 | 0.0004 | 0.0000 |

* Mean values only, no statistics
