# Supplementary material for: Carrageenan Gum and Adherent Invasive Escherichia coli in a Piglet Model of Inflammatory Bowel Disease: Impact on Intestinal Mucosa-associated Microbiota
Source: Front Microbiol. 2016 Apr 5;7:462. doi: 10.3389/fmicb.2016.00462 (PMC4820460; doi:10.3389/fmicb.2016.00462)
Supplement: Supplementary Table 5 — A summary showing mean relative abundances of taxa in the descending colon mucosa samples. [file Table5.docx]

| **Supplementary Table 5:** A summary showing mean relative abundances of taxa in the **Descending colon** **mucosa samples**. While majority of taxa were classified at the genus level (g.) some were only classified at the phylum (p.), class (c.), order (o.), or family (f.) levels. | | | | |
| --- | --- | --- | --- | --- |
| **Taxa** | **Mean relative abundance*** | | | |
|  | **Control** | **UM146** | **CG** | **CGUM146** |
| **-------------------------------Greater than or equal to 0.01% of community--------------------** | | | | |
| g. *Bifidobacterium* | 0.034 | 0.025 | 0.008 | 0.019 |
| p. Bacteroidetes | 0.031 | 0.029 | 0.017 | 0.011 |
| o. Bacteroidales | 11.058 | 14.144 | 5.353 | 7.271 |
| g. *Bacteroides* | 0.061 | 0.011 | 3.362 | 10.584 |
| f. Porphyromonadaceae | 0.095 | 0.004 | 0.005 | 0.001 |
| g. *Paludibacter* | 0.016 | 0.019 | 0.003 | 0.004 |
| g. *Parabacteroides* | 0.278 | 0.260 | 0.279 | 0.244 |
| g. *Prevotella* | 23.851 | 36.997 | 9.604 | 22.789 |
| f. S24-7 | 4.243 | 3.149 | 1.433 | 0.887 |
| g. *Butyricimonas* | 0.040 | 0.007 | 0.039 | 0.011 |
| g. *CF231* | 2.581 | 2.617 | 0.857 | 0.686 |
| g. *YRC22* | 0.021 | 0.008 | 0.012 | 0.011 |
| f. p-2534-18B5 | 0.088 | 0.011 | 0.004 | 0.006 |
| f. Chlamydiaceae | 0.005 | 0.040 | 0.007 | 0.000 |
| o. YS2 | 0.077 | 0.110 | 0.031 | 0.023 |
| g. *Mucispirillum* | 0.039 | 0.054 | 8.050 | 3.526 |
| p. Firmicutes | 0.123 | 0.164 | 0.161 | 0.103 |
| f. Lactobacillaceae | 0.109 | 0.065 | 0.176 | 0.400 |
| g. *Lactobacillus* | 7.729 | 9.847 | 10.233 | 8.983 |
| f. Streptococcaceae | 0.125 | 0.080 | 1.896 | 0.294 |
| g. *Streptococcus* | 0.017 | 0.028 | 0.562 | 0.212 |
| c. Clostridia | 0.398 | 0.293 | 0.152 | 0.533 |
| o. Clostridiales | 0.728 | 0.778 | 0.275 | 0.219 |
| f. Catabacteriaceae | 0.414 | 0.201 | 0.180 | 0.033 |
| f. Christensenellaceae | 0.018 | 0.019 | 0.005 | 0.008 |
| f. Clostridiaceae | 0.314 | 0.418 | 0.331 | 0.324 |
| g. *Clostridium* | 0.042 | 0.022 | 0.002 | 0.000 |
| g. *Sarcina* | 0.339 | 0.004 | 0.390 | 0.053 |
| f. Lachnospiraceae | 1.985 | 1.631 | 1.151 | 0.980 |
| g. *Blautia* | 1.462 | 0.808 | 0.607 | 0.361 |
| g. *Coprococcus* | 0.244 | 0.175 | 0.093 | 0.127 |
| g. *Dorea* | 0.255 | 0.247 | 0.231 | 0.152 |
| g. *Lachnobacterium* | 0.026 | 0.011 | 0.003 | 0.001 |
| g. *Lachnospira* | 0.071 | 0.076 | 0.016 | 0.038 |
| g. *Oribacterium* | 0.295 | 0.051 | 0.032 | 0.053 |
| g. *Roseburia* | 7.001 | 2.800 | 1.578 | 1.844 |
| g. *Peptococcus* | 0.039 | 0.028 | 0.026 | 0.010 |
| f. Peptostreptococcaceae | 0.041 | 0.016 | 0.040 | 0.002 |
| f. Ruminococcaceae | 7.654 | 5.369 | 2.419 | 2.248 |
| g. *Faecalibacterium* | 2.398 | 1.583 | 2.366 | 1.130 |
| g. *Oscillospira* | 3.156 | 2.988 | 0.923 | 1.661 |
| g. *Ruminococcus* | 2.362 | 2.350 | 0.924 | 2.540 |
| f. Veillonellaceae | 3.449 | 2.164 | 1.961 | 1.737 |
| g. *Acidaminococcus* | 0.348 | 0.166 | 0.270 | 0.099 |
| g. *Anaerovibrio* | 0.669 | 0.185 | 0.120 | 0.174 |
| g. *Dialister* | 3.729 | 0.768 | 1.171 | 1.017 |
| g. *Megasphaera* | 2.462 | 3.269 | 1.465 | 1.791 |
| g. *Mitsuokella* | 0.334 | 0.139 | 0.127 | 0.163 |
| g. *Phascolarctobacterium* | 0.296 | 0.240 | 0.394 | 0.091 |
| g. *Selenomonas* | 0.014 | 0.006 | 0.012 | 0.016 |
| f. Coriobacteriaceae | 0.125 | 0.014 | 0.035 | 0.021 |
| g. *Collinsella* | 0.025 | 0.005 | 0.031 | 0.010 |
| o. Erysipelotrichales | 0.033 | 0.016 | 0.006 | 0.005 |
| f. Erysipelotrichaceae | 0.292 | 0.142 | 0.250 | 0.135 |
| g. *Bulleidia* | 0.191 | 0.076 | 0.058 | 0.070 |
| g. *L7A_E11* | 0.041 | 0.009 | 0.002 | 0.001 |
| g. *p-75-a5* | 0.185 | 0.069 | 0.071 | 0.044 |
| g. *Catenibacterium* | 0.266 | 0.035 | 0.103 | 0.117 |
| o. ML615J-28 | 0.055 | 0.035 | 0.075 | 0.001 |
| f. Fusobacteriaceae | 0.000 | 0.000 | 0.020 | 0.101 |
| o. RF32 | 0.032 | 0.016 | 0.014 | 0.009 |
| g. *Sutterella* | 0.137 | 0.114 | 0.239 | 0.241 |
| g. *Oxalobacter* | 0.028 | 0.010 | 0.016 | 0.003 |
| g. *Ralstonia* | 0.038 | 0.015 | 0.004 | 0.002 |
| f. Desulfovibrionaceae | 0.002 | 0.003 | 0.022 | 0.019 |
| g. *Desulfovibrio* | 0.282 | 0.178 | 1.609 | 2.449 |
| o. GMD14H09 | 0.055 | 0.059 | 0.027 | 0.003 |
| o. Campylobacterales | 0.011 | 0.001 | 0.215 | 0.391 |
| g. *Campylobacter* | 2.305 | 0.905 | 6.545 | 9.437 |
| f. Helicobacteraceae | 0.002 | 0.000 | 0.040 | 0.008 |
| g. *Helicobacter* | 0.588 | 0.061 | 26.478 | 10.288 |
| f. Succinivibrionaceae | 0.012 | 0.022 | 0.164 | 0.909 |
| g. *Succinivibrio* | 0.253 | 0.634 | 0.257 | 0.124 |
| f. Enterobacteriaceae | 0.020 | 0.040 | 0.102 | 0.151 |
| g. *Escherichia* | 0.222 | 1.016 | 0.023 | 0.069 |
| f. Pasteurellaceae | 0.037 | 0.009 | 0.003 | 0.003 |
| f. Pseudomonadaceae | 0.117 | 0.081 | 0.041 | 0.048 |
| f. Xanthomonadaceae | 0.022 | 0.005 | 0.003 | 0.018 |
| g. *Treponema* | 0.628 | 0.465 | 2.993 | 0.271 |
| g. *Brachyspira* | 0.000 | 0.000 | 0.076 | 0.035 |
| c. Mollicutes | 0.022 | 0.009 | 0.005 | 0.005 |
| g. *RFN20* | 0.173 | 0.135 | 0.140 | 0.040 |
| g. *Mycoplasma* | 0.173 | 0.060 | 0.004 | 0.141 |
| o. RF39 | 1.619 | 0.481 | 0.131 | 0.759 |
| Unclassified | 0.482 | 0.595 | 0.637 | 0.410 |
| **--------------------------------------Less than 0.01%---------------------------------------------------** | | | | |
| g. *Corynebacterium* | 0.0024 | 0.0000 | 0.0000 | 0.0000 |
| f. BS11 | 0.0040 | 0.0013 | 0.0000 | 0.0137 |
| f. Bacteroidaceae | 0.0000 | 0.0000 | 0.0099 | 0.0230 |
| f. Marinilabiaceae | 0.0012 | 0.0000 | 0.0000 | 0.0000 |
| f. Prevotellaceae | 0.0011 | 0.0050 | 0.0009 | 0.0009 |
| f. Rikenellaceae | 0.0000 | 0.0011 | 0.0037 | 0.0067 |
| f. Chitinophagaceae | 0.0009 | 0.0016 | 0.0005 | 0.0000 |
| f. Flexibacteraceae | 0.0092 | 0.0000 | 0.0000 | 0.0000 |
| g. *Pedobacter* | 0.0012 | 0.0000 | 0.0089 | 0.0013 |
| o. CAB-I | 0.0005 | 0.0000 | 0.0000 | 0.0004 |
| o. Streptophyta | 0.0007 | 0.0000 | 0.0000 | 0.0012 |
| f. Elusimicrobiaceae | 0.0049 | 0.0016 | 0.0009 | 0.0000 |
| g. *Fibrobacter* | 0.0081 | 0.0037 | 0.0027 | 0.0010 |
| c. Bacilli | 0.0000 | 0.0000 | 0.0007 | 0.0013 |
| g. *Staphylococcus* | 0.0005 | 0.0258 | 0.0000 | 0.0000 |
| o. Lactobacillales | 0.0058 | 0.0085 | 0.0172 | 0.0069 |
| g. *Enterococcus* | 0.0000 | 0.0000 | 0.0000 | 0.0075 |
| g. *Dehalobacterium* | 0.0241 | 0.0084 | 0.0012 | 0.0010 |
| g. *Anaerofustis* | 0.0013 | 0.0006 | 0.0015 | 0.0000 |
| g. *Anaerostipes* | 0.0082 | 0.0030 | 0.0014 | 0.0027 |
| g. *Butyrivibrio* | 0.0076 | 0.0031 | 0.0000 | 0.0041 |
| g. *rc4-4* | 0.0015 | 0.0062 | 0.0000 | 0.0007 |
| g. *Anaerotruncus* | 0.0002 | 0.0008 | 0.0142 | 0.0005 |
| g. *Veillonella* | 0.0010 | 0.0000 | 0.0101 | 0.0217 |
| o. Coriobacteriales | 0.0204 | 0.0052 | 0.0053 | 0.0000 |
| g. *Slackia* | 0.0019 | 0.0000 | 0.0005 | 0.0004 |
| g. *Allobaculum* | 0.0000 | 0.0018 | 0.0000 | 0.0006 |
| o. Erysipelotrichales | 0.0004 | 0.0000 | 0.0000 | 0.0004 |
| g. *Sharpea* | 0.0023 | 0.0107 | 0.0005 | 0.0012 |
| f. Victivallaceae | 0.0025 | 0.0043 | 0.0007 | 0.0004 |
| f. R4-45B | 0.0000 | 0.0013 | 0.0000 | 0.0000 |
| f. Pirellulaceae | 0.0085 | 0.0128 | 0.0036 | 0.0025 |
| p. Proteobacteria | 0.0028 | 0.0008 | 0.0175 | 0.0194 |
| g. *Devosia* | 0.0071 | 0.0003 | 0.0000 | 0.0000 |
| g. *Hyphomicrobium* | 0.0018 | 0.0000 | 0.0000 | 0.0000 |
| g. *Sphingobium* | 0.0012 | 0.0000 | 0.0030 | 0.0000 |
| c. Betaproteobacteria | 0.0021 | 0.0000 | 0.0021 | 0.0073 |
| o. Burkholderiales | 0.0107 | 0.0044 | 0.0072 | 0.0022 |
| f. Alcaligenaceae | 0.0007 | 0.0003 | 0.0046 | 0.0002 |
| f. Comamonadaceae | 0.0085 | 0.0000 | 0.0000 | 0.0016 |
| f. Oxalobacteraceae | 0.0145 | 0.0000 | 0.0020 | 0.0044 |
| c. Deltaproteobacteria | 0.0019 | 0.0035 | 0.0026 | 0.0047 |
| o. Desulfovibrionales | 0.0000 | 0.0003 | 0.0005 | 0.0015 |
| f. Campylobacteraceae | 0.0003 | 0.0000 | 0.0155 | 0.0020 |
| c. Gammaproteobacteria | 0.0035 | 0.0066 | 0.0000 | 0.0102 |
| g. *Anaerobiospirillum* | 0.0000 | 0.0070 | 0.0138 | 0.0011 |
| g. *Acinetobacter* | 0.0062 | 0.0112 | 0.0047 | 0.0054 |
| g. *Alkanindiges* | 0.0010 | 0.0000 | 0.0006 | 0.0012 |
| g. *Enhydrobacter* | 0.0015 | 0.0015 | 0.0000 | 0.0000 |
| g. *Pseudomonas* | 0.0019 | 0.0000 | 0.0000 | 0.0015 |
| g. *Sphaerochaeta* | 0.0004 | 0.0008 | 0.0007 | 0.0000 |
| f. Sphaerochaetaceae | 0.0003 | 0.0008 | 0.0011 | 0.0000 |
| g. *Candidatus* *Cloacamonas* | 0.0004 | 0.0003 | 0.0000 | 0.0000 |
| f. Dethiosulfovibrionaceae | 0.0208 | 0.0049 | 0.0033 | 0.0000 |
| o. Anaeroplasmatales | 0.0048 | 0.0023 | 0.0013 | 0.0000 |
| f. Anaeroplasmataceae | 0.0005 | 0.0000 | 0.0000 | 0.0067 |
| f. RFP12 | 0.0066 | 0.0003 | 0.0003 | 0.0000 |

^*^Mean values only; no statistics.
